# Naphthoquinone derivatives as potential immunomodulators: prospective for COVID-19 treatment†

**DOI:** 10.1039/d3ra08173g

**Published:** 2024-02-21

**Authors:** Vitor Tassara Moraes, Franco Jazon Caires, Pedro V. da Silva-Neto, Jacqueline Nakau Mendonça, Thais F. C. Fraga-Silva, Bianca Bueno Fontanezi, Priscyla Daniely Marcato, Vania Luiza Deperon Bonato, Carlos Arterio Sorgi, Luiz Alberto Beraldo Moraes, Giuliano Cesar Clososki

**Affiliations:** a Departamento de Ciências Biomoleculares, Faculdade de Ciências Farmacêuticas de Ribeirão Preto, Universidade de São Paulo-USP Ribeirão Preto 14040-903 SP Brazil gclososki@usp.br +55 16 3315-4208; b Departamento de Química, Faculdade de Filosofia, Ciências e Letras de Ribeirão Preto-FFCLRP, Universidade de São Paulo-USP Ribeirão Preto 14040-901 SP Brazil; c Instituto de Ciências Biológicas e da Saúde, Universidade Federal de Alagoas-UFAL Maceió 57072-900 AL Brazil; d Departamento de Bioquímica e Imunologia, Faculdade de Medicina de Ribeirão Preto-FMRP, Universidade de São Paulo-USP Ribeirão Preto 14040-900 SP Brazil; e Departamento de Ciências Farmacêuticas, Faculdade de Ciências Farmacêuticas de Ribeirão Preto, Universidade de São Paulo-USP Ribeirão Preto 14040-903 SP Brazil

## Abstract

Inflammation plays a crucial role in COVID-19, and when it becomes dysregulated, it can lead to severe outcomes, including death. Naphthoquinones, a class of cyclic organic compounds widely distributed in nature, have attracted significant interest due to their potential biological benefits. One such naphthoquinone is 3,5,8-trihydroxy-6-methoxy-2-(5-oxohexa-1,3-dienyl)-naphthanthene-1,4-dione (3,5,8-TMON), a compound produced by fungi. Despite its structural similarity to shikonin, limited research has been conducted to investigate its biological properties. Therefore, the objective of this study was to evaluate the effects of 3,5,8-TMON and its synthetic derivatives in the context of inflammation induced by lipopolysaccharide (LPS) and SARS-CoV-2 infection *in vitro* using cell cultures. 3,5,8-TMON was obtained by acid treatment of crude extracts of fermentation medium from *Cordyceps* sp., and two derivatives were accessed by reaction with phenylhydrazine under different conditions. The results revealed that the crude extract of the fungi (C. Ex) inhibited the activity of transcription factor NF-kB, as well as the production of nitric oxide (NO) and interleukin-6 (IL-6) when LPS induced it in RAW 264.7 cells. This inhibitory effect was observed at effective concentrations of 12.5 and 3.12 μg mL^−1^. In parallel, 3,5,8-TMON and the new derivatives 3 and 4 demonstrated the ability to decrease IL-6 production while increasing TNF, with a specific effect depending on the concentration. These concentration-dependent agonist and antagonist effects were observed in THP-1 cells. Furthermore, 3,5,8-TMON inhibited IL-6 production at concentrations of 12.5 and 3.12 μg mL^−1^ in Calu-3 cells during SARS-CoV-2 viral infection. These findings present promising opportunities for further research into the therapeutic potential of this class of naphthoquinone in the management of inflammation and viral infections.

## Abstract 2

The microbial naphthoquinone 3,5,8-TMON and its derivatives showed great anti-inflammatory activity, regulating cytokines and with promising activity mitigating COVID-19 inflammatory impacts.

## Graphical abstract

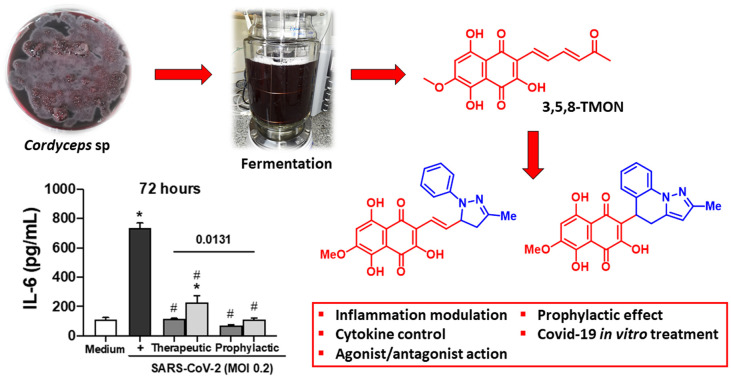

## 1 Introduction

Inflammation is a multifaceted response initiated by the human body's defense system in response to noxious stimuli. These stimuli can include foreign bodies, pathological or nonpathological microorganisms, and tissue injuries.^1^ This complex process involves specific cells, receptors, and mediators. When properly regulated, inflammation plays a pivotal role in assisting the host in combatting parasites or infectious agents. However, loss of control over this process can result in pathological inflammation, disrupting homeostasis and potentially transforming a patient with a minor infection into a critically ill individual, as observed in conditions like sepsis.^2^ Moreover, during the COVID-19 pandemic, a prominent feature has been the development of hyper-inflammation in some infected individuals. This hyper-inflammatory response, characterized by excessive cytokine release, can contribute to severe respiratory distress and multi-organ dysfunction, highlighting the critical importance of understanding and managing inflammation in the context of emerging infectious diseases like COVID-19.^3,4^

In the context of a general inflammatory process, the sequence of events unfolds as follows: initially, specific receptors known as pattern-recognition receptors (PRR) within particular cell types recognize inducers referred to as pathogen-associated molecular patterns (PAMP).^5^ Subsequently, depending on the inducers encountered, these cells initiate the production of various inflammatory mediators, which can be categorized into distinct groups characterized by their functions and biochemical properties. These groups encompass vasoactive amines (such as histamine), vasoactive peptides (including substance P), fragments of complement compounds (*e.g.*, C3_a_), lipid mediators (like prostaglandins), cytokines (such as TNF and IL-6), chemokines, and proteolytic enzymes (such as elastin).^2^ These mediators interact with cells and tissues, modifying their functional states. It's important to note that each cell and tissue responds differently to each mediator. These responsive cells, known as effectors, bear the responsibility of eliminating the underlying cause of the inflammation.

**Fig. 1:**
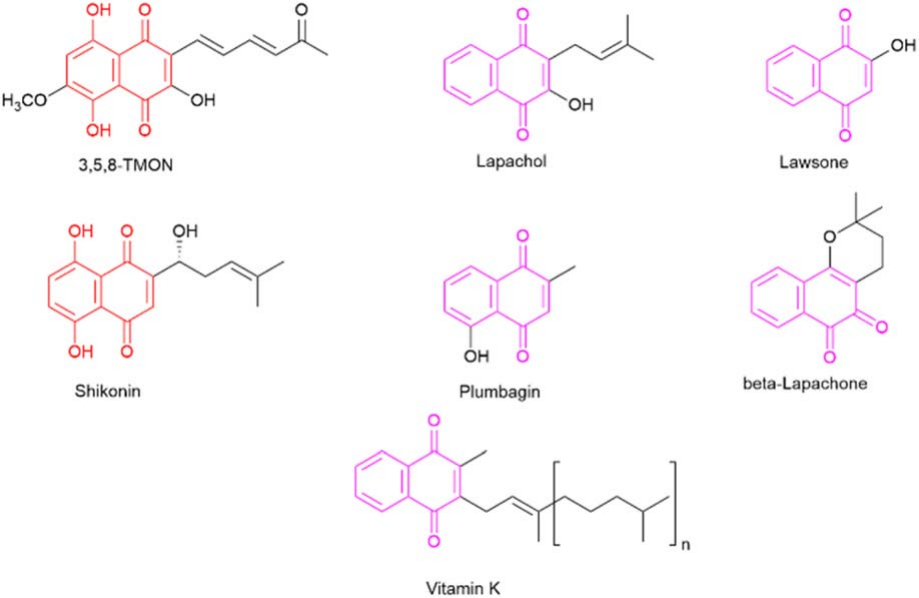
Representative naphthoquinone-based important compounds.

The desire to alleviate inflammation has been a long-standing pursuit for humanity. This quest dates back to the historical use of medicinal plants containing salicylates and extends to the contemporary widespread use of aspirin and NSAIDs.^6^ Despite the diverse array of drugs available on the market, including glucocorticoids, there remains a pressing need to develop more effective and less toxic agents to combat inflammation.^7^ In this context, specific classes of natural products have attracted significant attention for their potential to inhibit the inflammatory process. One noteworthy class is naphthoquinones, which are characterized by a naphthalene skeleton and typically possess two carbonyl groups, positioned either at C-1 and C-4 or at C-1 and C-2 within their structure. Additionally, these molecules exhibit a wide array of substituent groups that can be attached to different structural positions. Naphthoquinones play an essential role in various organisms, including humans, where vitamin K is crucial to maintaining biological processes.^8^ Several naphthoquinones have been investigated for their biological activities, encompassing antimalarial,^9^ anti-cancer,^10,11^ antimycobacterial,^12,13^ and anti-inflammatory properties.^14^ Notable examples of plant-derived naphthoquinones used in biological activity assessments include lawsone, lapachol, β-lapachone, plumbagin, and shikonin (see Fig. 1). Among these, plumbagin and particularly shikonin have demonstrated utility as anti-inflammatory agents.^7,15^

Shikonin, a naphthoquinone found in the roots of *Lithospermum erythrorhizon* Siebold & Zucc,^16^ has attracted attention for its therapeutic properties.^14^ However, the overexploitation of shikonin-producing plants has raised environmental concerns.^15^ In contrast, 3,5,8-trihydroxy-6-methoxy-2-(5-oxohexa-1,3-dienyl)-naphthanthene-1,4-dione (3,5,8-TMON, 2) is a naturally occurring naphthoquinone produced by the fungus *Cordyceps* sp.^17^ It shares a striking structural similarity with shikonin, characterized by two hydroxyl groups on the naphthoquinone ring, also known as the naphthazarin group. The fungal production of 3,5,8-TMON provides an environmentally friendly alternative, as it can be effectively cultivated in bioreactors, thus helping to reduce its environmental footprint. The structural resemblance between 3,5,8-TMON and shikonin has piqued our interest in exploring the potential biological activities associated with this compound.^18^ Given the limited information available about the biological properties of this compound and its derivatives, this study aims at structurally modifying the unexplored naphthoquinone 3,5,8-TMON. In this study, we synthesize new compounds and assess their biological activity, particularly in inhibiting innate immune cell production of inflammatory mediators, as in the case of SARS-CoV-2 infection *in vitro*.

## 2 Experimental section

### 2.1. Materials, methods and equipment

^1^H NMR, ^13^C NMR, DEPT, ^1^H–^1^H COSY, HMQC and HMBC spectra were recorded on a Bruker® Avance III HD-600 for the synthetic products (operating at 600 MHz for proton and 150 MHz for carbon) and on a Bruker® DRX-400 for the naphthoquinone (operating at 400.1 MHz for proton and 100.6 MHz for carbon) for the natural products. ESI+ HRMS spectra were obtained from a Triple-TOF 5600 Sciex mass spectrometer coupled with a HPLC Nexera system. Microwave assisted reactions were carried out using a Monowave™ 300 – Anton Paar.

### 2.2. General laboratory procedures

#### 2.2.1. Analytical methods

The reactions were monitored by LC-DAD-MS, employing a liquid chromatograph coupled with a mass spectrometer Acquity Xevo TQS (Waters®), Ascentis® Express C18 column (10.0 cm × 3 mm, 2.7 μm) from Sigma-Aldrich. The setup included a DAD detector (200–700 nm) and both analysis modes, positive and negative, were employed to identify the reaction products.

For preparative High Performance Liquid Chromatography, a Shimadzu® HPLC system, equipped with CBM-20A controller, LC6AD bombs, SPD-20A detector, DGU-20A5 degas and Rheodyne manual injector, was used. Initially, a Shimadzu-ODS C18 column (250 × 4.6 mm, 5 μm) was used, followed by a Luna C18 column (250 × 20.00 mm, 5 μm) from Phenomenex®. The method was a linear gradient, mixture of methanol and water, both with 0.1% of formic acid, starting from the proportion 60 : 40 (MeOH/H_2_O) to 98 (MeOH/H_2_O) in 30 minutes. The flow rate was set at 1 mL min^−1^ for the analytic mode and 12 mL min^−1^ for preparative mode. The wavelength range used was 371–480 nm.

#### 2.2.2. Cell culture and anti-inflammatory assay *in vitro*

The Raw Blue™ [264.7] macrophages (INVIVOGEN) were cultured in DMEM medium supplemented with Normocin (50 mg mL^−1^), Zeocin (25 mg mL^−1^) and FBS (10%) and used for cytotoxicity and anti-inflammatory activity determination. For the cytotoxicity assay, a cell suspension (2.5 × 10^5^ cells per well) were seeded in 48-well plate and kept overnight at 37 °C and 5% CO_2_. In the following day, cells were treated with different concentrations (6.25–100 μg mL^−1^) of naphthoquinone. After 24 h, the medium was removed and the solution containing Resazurin dye (40 μM) was added and incubated for 3 hours at the same experimental conditions. Afterwards, the fluorescence was read in a microplate reader at an excitation wavelength of 560 nm and emission of 590 nm. Cell viability was achieved by comparing the percentage between the cells treated with the negative control, just medium. On the other hand, DMSO 10% was used as positive control.

The anti-inflammatory studies were performed in one plate pre-incubated for 2 hours with the naphthoquinones treatments (prophylactic), while another plate was stimulated for 2 hours with LPS (0.5 mg mL^−1^) from *E. coli*, for the therapeutic treatment. After the initial incubation time, LPS (0.5 mg mL^−1^) was added to the prophylactic plate and the naphthoquinone treatments were added to the therapeutic plate. The exposure time of LPS was standardized for both treatment methods for total 24 hours. After the incubation period, the supernatants were removed from the wells and reserved.

NO and its nitrite metabolite (NO_2_) may react with sulfanilamide in an acid medium by the colorimetric method Griess. The reaction produces a diazo compound that turns pink. Griess's reagent 1 : 1 (v : v) was prepared by 1% sulfanilamide and 0.1% *N*-(1-naphthyl)-ethylenediamine (NED). Then, the supernatant reserved from the sample was added to Griess reagent (1 : 1 v/v) in a 96-well plate. After 5 minutes of reaction, the plate was read in a microplate reader at 560 nm (Quant, Biotek Instruments Inc., Winooski, VT). A calibration curve was made with a standard sodium nitrite solution with concentrations from 0.391 to 200 μM as a calibration curve.

Raw-Blue™ stably expresses the SEAP gene (secreted embryonic alkaline phosphatase) inducible by NF-κB transcription factors, which is measured using QUANTI-Blue™ (Invivogen). For that, in 96-well plate, 20 μL of the supernatant of BRP extract, ACET-FR and DICL-FR in the maximum activity concentration was incubated with reagent for 1 h and then the absorbance was measured at 650 nm by ELISA reader (Quant, Biotek Instruments Inc., Winooski, VT). The percentage of NF-κB reduction was calculated using LPS control as 100%.

Human adenocarcinomic lung epithelial (Calu-3, BCRJ 0264) and human peripheral blood monocyte (THP-1, BCRJ 0234) cell lines were maintained at 37 °C, 5% CO_2_ in DMEM supplemented with 20% FBS and 1% penicillin/streptomycin. For SARS-CoV-2 infection, Calu-3 cells were infected at MOI 0.2 in DMEM supplemented with 2% FBS, l-glutamine, non-essential amino acids, and sodium pyruvate. All cultures were maintained at 37 °C, 5% CO_2_.

#### 2.2.3. Quantification of cytokine

The supernatants were used for quantification of cytokines (IL 6, TNF) by Elisa (R&D Systems, Minnesota, mn, USA), according to the manufacturer's instructions.

#### 2.2.4. Statistical analysis

Statistical analysis was performed using the GraphPad Prism software (GrandPad Software Inc., San Diego, CA, USA) by ANOVA one-way with multiple Tukey's comparisons.

### 2.3. Experimental procedures and data

#### 2.3.1. General procedure to obtain 3,5,8-TMON (2)

To obtain the 3,5,8-TMON, we followed the procedures found in the literature with a few modifications.^17^*Cordyceps* sp. was cultivated in a potato dextrose liquid broth culture Erlenmeyer, on a rotatory shaker (200 rpm), for 15 days at room temperature (25 °C). After that, the culture was extracted with ethyl acetate 1 : 3 ratio at pH 2 with *o*-phosphoric acid. The combined organic fractions were dried with anhydrous MgSO_4_, filtered, and then concentrated under vacuum. Next, 500 mL of H_2_O and 3 mL of *o*-phosphoric acid were added to the solid, and the mixture was heated to reflux overnight. After that, the pH was adjusted to 7 with NaOH and the solution cooled down at the refrigerator. The precipitate was separated with centrifugation at 12 000*g*. The solid was dried at room temperature with ventilation, and a small fraction was dissolved with MeOH for LC-MS analysis to confirm the purity of the material. The structure was confirmed by NMR (^1^H and ^13^C), operating at 400 MHz for hydrogens.

#### 2.3.2. General procedure for synthesis of compounds 3 and 4

Phenyl hydrazine hydrochloride (0.197 mmol) was added to a solution of 0.151 mmol of 3,5,8-TMON in 15 mL of acetic acid. The mixture was heated, either refluxed in a conventional reflux system or in a microwave reactor (closed vessel), for 1 hour. After that, the reaction was extracted with ethyl acetate and concentrated under vacuum. A red solid was obtained and analyzed at LC-MS.

#### 2.3.3. Experimental data

##### 2.3.3.1. Erythrostominone (1)

*δ*_H_ (500 MHz, CDCl_3_): 13.15 (1H, s, phenolic OH), 12.58 (1H, s, phenolic OH), 6.**35** (1H, s, H-7), 4.9**0** (1H, dd, *J* = **4.2** and 1.**9** Hz, H-4), 4.7**1** (1H, m, H-2), 3.88 (3H, s, 8-OCH_3_), 3.06 (1H, dd, *J* = 16.8 and 7.1 H_a_-1), 2.76 (1H, dd, *J* = 16.8 and 5.3 Hz, H_b_-1), 2.23 (3H, s, –CH_3_), 2.16 (1H, dt, *J* = 14.5 and 2.0 Hz, H_a_-3), 1.72 (1H, ddd, *J* = 14.5; 12.3 and 4.1 Hz, H_b_-3). ESI+ CID: 349 (22), 331 (8), 313 (12), 291 (13), 273 (100).

##### 2.3.3.2. 3,5,8-Trihydroxy-6-methoxy-2-(5-oxohexa-1,3-dienyl)-1,4-naphthoquinone (2)

*δ*_H_ (400 MHz, DMSO-d_6_): 14.41 (1H, s, phenolic OH), 12.50 (1H, s, phenolic OH), 7.65 (1H, dd, *J* = 15.4 and 11.2 Hz, H-2′), 7.38 (1H, dd, *J* = 15.4 and 11.3 Hz, H-3′), 7.28 (1H, d, *J* = 15.4 Hz, H-1′), 6.79 (1H, s, H-7), 6.07 (1H, d, *J* = 15.4 Hz, H-4′), 3.88 (3H, s, O–CH_3_), 2.24 (3H, s, –CH_3_). ESI+ CID (30 eV, N2): 289.0727 (35), 273.0407 (43), 249.0413 (57), 231.0302 (35), 221.0464 (100), 206.0217 (73), 193.0502 (73).

##### 2.3.3.3. (*E*) 3,5,8-Trihydroxy-6-methoxy-2-(2-(3-methyl-1-phenyl-4,5-dihydro-1*H*-pyrazol-5-yl)vinyl)-1,4-naphthoquinone (3)

*δ*_H_ (600 MHz; CDCl_3_): 13.38 (1H, s, phenolic OH), 12.05 (1H, s, phenolic OH), 7.23 (2H, t, *J* = 7.8 Hz, H-(*m*)aromatic), 7.18 (1H, dd, *J* = 16.3 and 7.6 Hz, H-2′), 7.13 (2H, d, *J* = 8.1 Hz, H-(*o*)-aromatic), 6.92 (1H, d, *J* = 16.4 Hz, H-1′), 6.82 (1H, t, *J* = 7.3 Hz, H-(*p*)aromatic), 6.62 (1H, s, H-7), 4.71 (1H, dt, *J* = 11.5 and 7.9 Hz, H-5′′), 3.98 (3H, s, O–CH_3_), 3.25 (1H, dd, *J* = 17.4 and 11.4 Hz, H_b_-4′′), 2.77 (1H, dd, *J* = 17.4 and 8.2 Hz, H_a_-4′′), 2.09 (3H, s, –CH_3_). ESI+ CID (30 eV, N2): 421.1431 (52), 171.0945 (100).

##### 2.3.3.4. (*E*) 3,5,8-Trihydroxy-6-methoxy-2-(2-(3-methyl-1-phenyl-4,5-dihydro-1*H*-pyrazol-5-yl)vinyl)-1,4-naphthoquinone (4)

*δ*_H_ (600 MHz; CDCl_3_): 13.22 (1H, s, phenolic OH), 11.96 (1H, s, phenolic OH), 7.85 (1H, d, *J* = 8 Hz, H-9′), 7.24 (1H, t, *J* = 7.7 Hz, H-8′), 6.96 (1H, t, *J* = 7.5 Hz, H-7′), 6.88 (1H, d, *J* = 7.6 Hz, H-6′), 6.55 (1H, s, H-7), 5.88 (1H, s, H-3′), 4.82 (1H, dd, *J* = 12.1 and 6.4 Hz, H-5′), 3.92 (3H, s, O–CH_3_), 3.45 (1H, dd, *J* = 15.5 and 12.2 Hz, H_b_-4′), 3.02 (1H, dd, *J* = 15.5 and 6.4 Hz, H_a_-4′), 2.29 (3H, s, –CH_3_). ESI+ CID (30 eV, N2): 419.1269 (100), 404.1033 (60), 401.1159 (52), 391.1319 (77), 375.1007 (87), 358.0973 (84), 317.0708 (41), 183.0932 (92).

## 3 Results and discussion

### 3.1. Obtaining 3,5,8-TMON

**Fig. 2:**
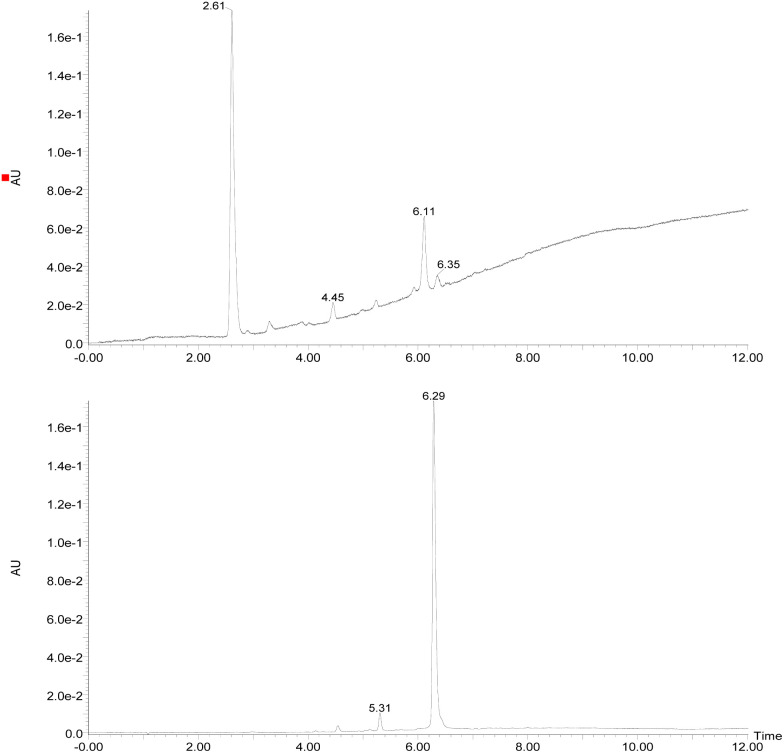
Chromatogram DAD (240–700 nm) (top of the image) obtained from the crude extract of the *Cordyceps* sp. and (bottom of the image) obtained after dehydration reaction of the crude extract. (RT = 2.61 min (erythrostominone) and RT = 6.29 min (3,5,8-TMON)).

Initially, the fungus *Cordyceps* sp. was grown in an Erlenmeyer flask with potato-dextrose liquid culture for 15 days. After that, ethyl acetate was used to extract the organic components of the mixture; then, the combined organic fractions were dried under vacuum. At the end of the procedure, we obtained a red solid that we called the crude extract (C. Ex). This crude extract was analyzed by LC-MS (Fig. 2), and the main compounds were identified as erythrostominone (1) and 3,5,8-TMON (2), the second as a minority product, which corresponds to the literature data.^17^ A sort of crude extract was separated from bioactivity experiments and the rest was used to obtain pure naphthoquinone.

**Scheme 1:**
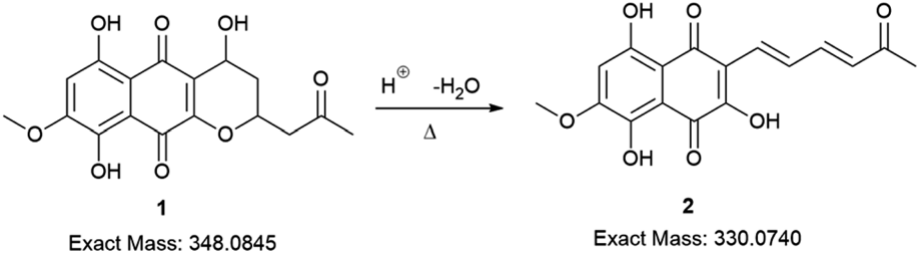
Conversion of erythrostominone to 3,5,8-TMON. Conditions: aqueous solution of 1, 3 mL of *o*-phosphoric acid, reflux overnight.

The red solid obtained from liquid–liquid extraction with AcOEt (crude extract) was added to a flask with water and *o*-phosphoric acid at pH 3 and heated to reflux overnight. During this procedure, compound 1 had its dihydropyran ring opened and underwent dehydration, resulting in complete conversion to compound 2 (Scheme 1).^17,18^ After cooling, the mixture was centrifuged and the solid was separated and analyzed by LC-MS, confirming the complete conversion of the metabolites and the expected purity of the final product^17^ (Fig. 2). HRMS and NMR spectra were also obtained to confirm the structure and purity of the product. Compound 2 was obtained as a red solid and then used a starting material for the preparation of novel naphthoquinone derivatives.

### 3.2. Modification reactions

**Scheme 2:**
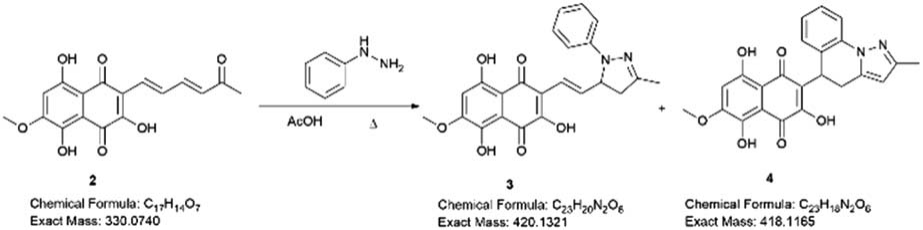
Structural modification of 3,5,8-TMON. Conditions: 2 (1 eq.), phenyl hydrazine hydrochloride (1.3 eq.), AcOH (15 mL), reflux temperature (oil bath or microwave). Isolated yields: 3 (15–20%) and 4 (0–25%).

Examining the structure of 3,5,8-TMON, we identified electrophilic sites prone to nucleophilic addition reactions, particularly within the polyconjugate system with α, β, γ, δ – unsaturation conjugated to a ketone. This system offers the potential for the formation of various heterocycles, including dihydropyrazoles, by utilizing phenyl hydrazine reactants.^19^ Moreover, the carbonyl sites can give access to hydrazone derivatives.^20^ Therefore, we initiated derivatization studies by reacting 3,5,8-TMON with phenyl hydrazine in acetic acid as solvent, either under heating in an oil bath or using a microwave reactor (Scheme 2).

**Fig. 3:**
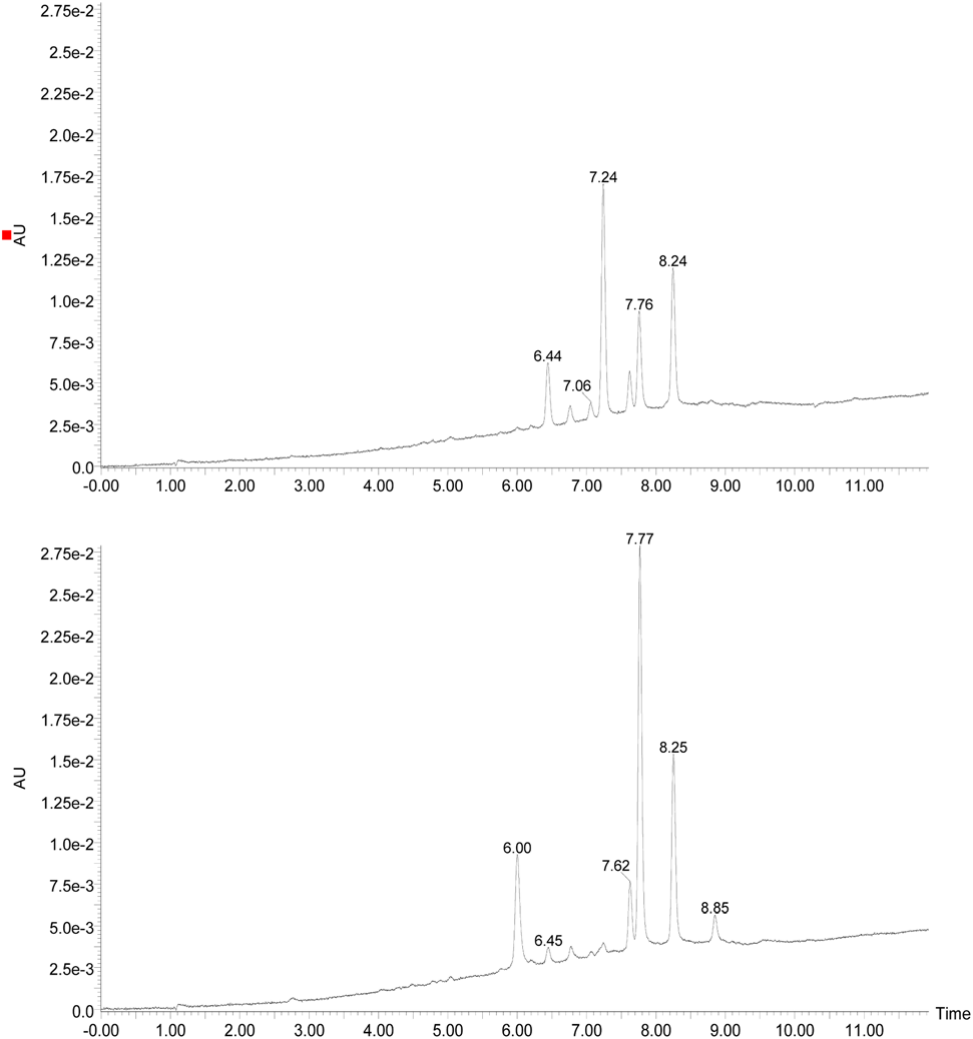
Chromatogram DAD (240–700 nm) obtained from the reaction between 3,5,8-TMON and phenyl hydrazine in AcOH employing oil bath (top of the image) and microwave (bottom of the image). (RT = 7.24 min (4), RT = 7.77 (3) and RT = 8.25 (5)).

The reactions were monitored by LC-MS, and the generation of possible products of interest was observed within one hour of the reaction. There were three major products of interest, which were named as follows: deriv. 1 (3) (r.t.: 7.77) min [M + H]^+^ of *m*/*z* 421 and [M − H]^−^ of *m*/*z* 419, deriv. 2 (4) (r.t.: 7.24 min) [M + H]^+^ of *m*/*z* 419 and [M − H]^−^ of *m*/*z* 417 and deriv. 3 (5) (r.t.: 8.25) [M + H]^+^ of *m*/*z* 419 and [M − H]^−^ of *m*/*z* 417. Analysing the product masses, we can reasonably assume that 3 (with a mass of 420 u) is generated through the condensation between phenyl hydrazine and the naphthoquinone. In contrast, 4 and 5 (both with a mass of 418 u) likely correspond to the oxidized product derivatives (Fig. 3).

Interestingly, different heating methods induce the generation of varying amounts of these products. For instance, the oil bath seemed to favour the generation of 4 rather than 3 and 5. In contrast, micro-wave heating favoured the formation of 3 while 4 was not detected (Fig. 3). Furthermore, after one hour in the microwave reactor, the naphthoquinone signal remained. These differences can be attributed to the effects of the microwave heating mechanisms, including the elimination of wall effects caused by inverted temperature gradients, and superheating effects of solvents at atmospheric pressure, within other unique microwave effects.^21^

Hydrazine derivatives have been already employed for the derivatization of naphthoquinones under acidic conditions.^20^ In addition, there exists a range of examples where α,β-unsaturated compounds, such as chalcones, are utilized in the synthesis of five-membered ring azaheterocycles.^19,22^ In the case of the 3,4,5-TMON system, its reaction with phenyl hydrazine appeared to favour the formation of the heterocycle through nucleophilic substitution at the side chain carbonyl, followed by an aza-Michael addition. Additionally, we observed an interesting cyclization involving the phenyl-hydrazine moiety and the side chain, resulting in the generation of a unique system attached to the naphthoquinone scaffold with three fused rings. A plausible mechanism for this reaction involves the generation of a stable carbocation at the side chain of the naphthoquinone, facilitating an attack from the *ortho* position of the phenyl ring, which is activated by its nitrogen donor character (see Fig. S2†).

The products were separated using preparative HPLC. The structures of deriv. 1 (3) and 2 (4) (Scheme 2) were determined through techniques such as ^1^H and ^13^C NMR, two-dimension NMR and high-resolution mass spectra (electrospray ionization). The structure of 5 was not proposed due to its poor solubility in the tested solvents.

Compound 3 was initially analyzed using high-resolution (electrospray ionization) mass spectrometry, which provided an *m*/*z* 421.1421 ion, corresponding to the molecular formula C_23_H_20_N_2_O_6_ and an accurate mass of 420.1321 u, with a 5.2 ppm error. The fragmentation spectra revealed one product ion of *m*/*z* 171, which we propose corresponds to the product resulting from the loss of the naphthoquinone ring and one methyl group from the side chain (Fig. S15†). The structure was fully assigned using NMR 2D analysis.

**Fig. 4:**
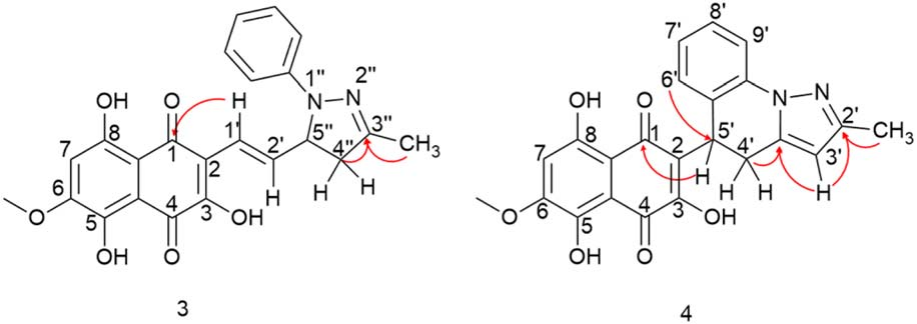
HMBC correlations of compounds 3 and 4.

The presence of a CH_2_ at C-4′′ position was identified through HMBC correlations (Fig. 4) between the protons H_a_-4′′ at *δ* 2.77 ppm and H_b_-4′′ at *δ* 3.26 ppm and the carbon from the imine group at *δ* 149.7 ppm (C-3′′). Additionally, a geminal hydrogen coupling (*J* = 17.4 Hz) was also observed for both H_a_-4′′ and H_b_-4′′. The presence of a CH_2_ group indicates a cyclization, as the hydrogen deficiency index of the molecule remains unchanged, indicating that there is no reduction reaction (HDI = 15). The formation of the imine group was confirmed by HMBC correlation between the methyl protons (3′′-CH_3_) at *δ* 2.10 ppm and the carbon at *δ* 149.7 ppm (C-3′′). This correlation was significant in confirming the reaction at the side chain. If this group were correlated with a carbonyl, it would result in a carbon signal at approximately *δ* 200 ppm. In contrast, the 3,5,8-TMON spectrum showed a carbon signal at *δ* 198.42 ppm.

Through HMBC correlations between proton H-1′ at *δ* 6.92 ppm and the naphthoquinone carbonyl group C-1 at *δ* 184.35 ppm, we were able to identify the first carbon in the side chain (C-1′ at *δ* 119.39 ppm). Moreover, a coupling constant of *J* = 16.4 Hz between proton H-1′ and H-2′ at *δ* 7.19 ppm confirmed the *trans* configuration of the double bound (Table 1).

**Table 1:** NMR spectra data for compound 3

C	*δ* ^1^H (ppm)	*J* (Hz)	*I*	*δ* ^13^C (ppm)	HMBC
1	—	—	—	184.35	—
2	—	—	—	119.23	—
3	—	—	—	152.14	—
4	—	—	—	179.91	—
4a	—	—	—	110.18	—
5C	—	—	—	161.99	—
6	—	—	—	156.69	—
7	6.63 (s)	—	1	108.81	C8_a_; C8; C5
8	—	—	—	152.44	—
8a	—	—	—	103.75	—
1′	6.92 (d)	16.4	1	119.39	C1; C3; C2′′ C5′′
2′	7.19 (dd)	16.4, 7.9	1	142.37	C1′; C5′′
3′′	—	—	—	149.7	—
4′′H_a_	2.77 (dd)	17.4, 7.9	1	45.19	C5′′; C3′′
4′′H_b_	3.26 (dd)	17.4, 11.5	1	45.19	C2′; C3′′
5′′	4.71 (dt)	11.5, 7.9	1	64.78	C1′; C4′′
Ar(*o*)	7.13 (d)	7.8	2	113.77	Ar(*p*); Ar(*o*)
Ar(*m*)	7.23 (t)	7.8	2	128.86	Ar; Ar(*m*)
Ar(*p*)	6.82 (t)	7.8	1	119.39	Ar(*o*)
N–Ar	—	—	—	147.01	—
6-OMe	3.99 (s)	—	3	56.61	C6
3′′-CH_3_	2.10 (s)	—	3	16.01	C3′′; C4′′
5-OH	12.05 (s)	—	1	—	C6
8-OH	13.38(s)	—	1	—	C8_a_; C7

The structure of compound 4 was assigned using a high-resolution mass spectrometer (electrospray ionization), which provided an ion with *m*/*z* 419.1260, corresponding to the molecular formula C_23_H_18_N_2_O_6_ and a mass of 418.1165 u, with a 4.1 ppm error. The fragmentation spectra revealed product ions with *m*/*z* 401 and 391, which were attributed to water (18 u) and CO (28 u) losses, respectively. Another product ion with *m*/*z* 183, was attributed to the complete loss of the naphthoquinone ring (236 u). The structure was fully confirmed using NMR 2D analysis (Table 2).

Aromatization of the five-membered ring was confirmed by a HMBC correlation between the H-3′ proton at *δ* 5.88 ppm and a C=N group at *δ* 150.03 ppm. Additionally, a HMBC correlation was observed between this proton and a quaternary carbon at *δ* 138.03 ppm (C-3′a). The position of the C=N group was further confirmed by the HMBC correlation between the C-2′ (*δ* 150.03 ppm) and CH_3_ protons at *δ* 2.29 ppm. HMBC correlations between the H-5′ at *δ* 4.82 ppm and the carbonyl group at *δ* 181.94 ppm (C-1) allowed for the confirmation that the first carbon in the side chain is the C-5′ at *δ* 31.28 ppm. This hydrogen exhibited two coupling constants, *J* = 12.1 and 6.4 Hz, indicating coupling with both hydrogens of the CH_2_ group in C-4′. Hydrogens H_a_-4′ and H_a_-4′, observed at *δ* 3.02 and 3.45 ppm, respectively, showed couplings with H-5′ and with each other, with a germinal coupling constant of *J* = 15.5 Hz. Finally, the HMBC correlations between these protons and carbon at *δ* 138.03 ppm (C-3′a) confirmed the position of the CH_2_ group. An HMBC correlation between the H-6′ at *δ* 6.88 and the C-5′ at *δ* 31.28 ppm was crucial in confirming the formation of the 6-membered ring, leading to the structural assignment of compound 4.

Both compounds 3 and 4, representing novel structures, were evaluated for biological activity, along with the crude extract of *Cordyceps* sp. (1) and compound 2.

**Table 2:** NMR spectra data for compound 4

C	*δ* ^1^H (ppm)	*J* (Hz)	*I*	*δ* ^13^C (ppm)	HMBC
1	—	—	—	181.94	—
2	—	—	—	125.16	—
3	—	—	—	154.27	—
4	—	—	—	177.82	—
4a	—	—	—	110.34	—
5	—	—	—	163.72	—
6	—	—	—	156.65	—
7	6.55 (s)	—	1	108.79	C5; C6; C8
8	—	—	—	155.23	—
8a	—	—	—	103.75	—
2′	—	—	—	150.03	—
3′	5.88 (s)	—	1	103.92	C2′; C3′a
3′a	—	—	—	138.03	—
4′H_a_	3.02 (dd)	15.5, 6.4	1	25.14	C2; C5′; C3′a
4′H_b_	3.45 (dd)	15.5, 12.2	1	25.14	C2; C5′; C3′a
5′	4.82 (dd)	12.2, 6.4	1	31.28	C1; C2; C3; C4′
5′a	—	—	—	125.83	—
6′	6.88 (d)	7.7	1	126.50	C8′; C9′a; C5′
7′	6.96 (t)	7.7	1	124.49	C5′a; C9′
8′	7.24 (t)	7.7	1	127.89	C6′; C9′a
9′	7.85 (d)	7.7	1	115.71	C7′; C9′a
9′a	—	—	—	136.25	—
6-OMe	3.92 (s)	—	3	56.59	C6
2′-CH_3_	2.29 (s)	—	3	13.69	C2′
5-OH	13.22 (s)	—	1	—	—
8-OH	11.96 (s)	—	1	—	C7; C8_a_

### 3.3. Assessing the cytotoxic and anti-inflammatory properties of naphthoquinones and their novel derivatives

**Fig. 5:**
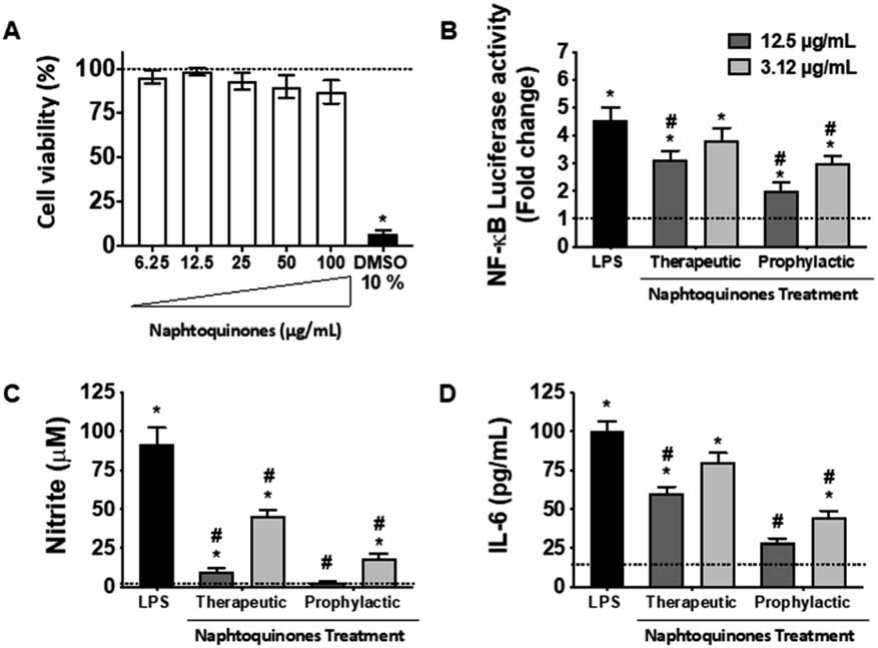
Effect of the crude extract on LPS-induced NF-κB transcriptional activity and inflammation response in RAW 264.7 cells. (A) Cell viability was measured by the MTT assay. (B) Cells were co-transfected with pNF-κB-Luc reporter and then were pretreated with the indicated concentrations of naphthoquinones for 2 h before (prophylactic) and 2 h after (therapeutic) LPS (1 μg mL^−1^) stimulation and cells were further incubated for 24 h. Cells were harvested and then the luciferase activities were determined by using the dual luciferase report assay system. (C) The nitrite production was measured by the Griess reaction. (D) The IL-6 concentration was determined using an ELISA kit. Crude extract treatment concetration was 3.12 μg mL^−1^ (light gray bars) and 12.5 μg mL^−1^ (dark gray bars). Values were presented as mean ± SEM of three independent experiments, *n* = 3 per experiment. *p < 0.05 *vs.* medium group and #*p* < 0.05 *vs.* LPS group.

In our investigation, we conducted an in-depth analysis of the crude extract to evaluate its potential as an inhibitor of lipopolysaccharide (LPS)-induced inflammatory mediator production in the murine macrophage cell line (RAW 264.7 cells). Notably, we observed minimal toxicity towards these macrophage cells at concentrations up to 100 μg mL^−1^ (Fig. 5A). Crucially, our findings revealed a substantial decrease in NF-kB activity (Fig. 5B) and inhibition of nitric oxide (NO) (Fig. 5C) and interleukin-6 (IL-6) (Fig. 5D) production at doses of 3.12 and 12.5 μg mL^−1^, indicating the anti-inflammatory potential of the crude extract. However, a notable observation emerged from our study, the anti-inflammatory effects of the 1,4-naphthoquinone crude extract employed herein appeared to be more pronounced in prophylactic treatments compared to therapeutic treatments *in vitro*.

This intriguing observation prompts several considerations, suggesting that the crude extract compounds may be particularly effective in preventing the initiation of the inflammatory response rather than halting it once underway. This preventive aspect could have implications for its use as a potential therapeutic agent in conditions characterized by chronic inflammation, where early intervention may be key. Our results also underscore the importance of dose and treatment timing in modulating the compound's anti-inflammatory effects. Further investigations should explore the underlying mechanisms and signaling pathways involved in this effect, but the NF-kB pathway was essentially involved, which sheds light on the mode of action. Considering the established anti-inflammatory effects of shikonin during the inflammatory process, its mechanisms of action primarily revolve around inhibiting the release of tumor necrosis factor (TNF) by modulating the NF-κB/TNF pathway. Furthermore, shikonin demonstrates the capacity to reduce the expression of other key inflammatory cytokines, IL-6 and IL-12. Additionally, this molecule exerts inhibitory effects on cyclooxygenase-2 (COX-2) expression, thus reducing prostaglandin production as a consequential effect.^23^

**Fig. 6:**
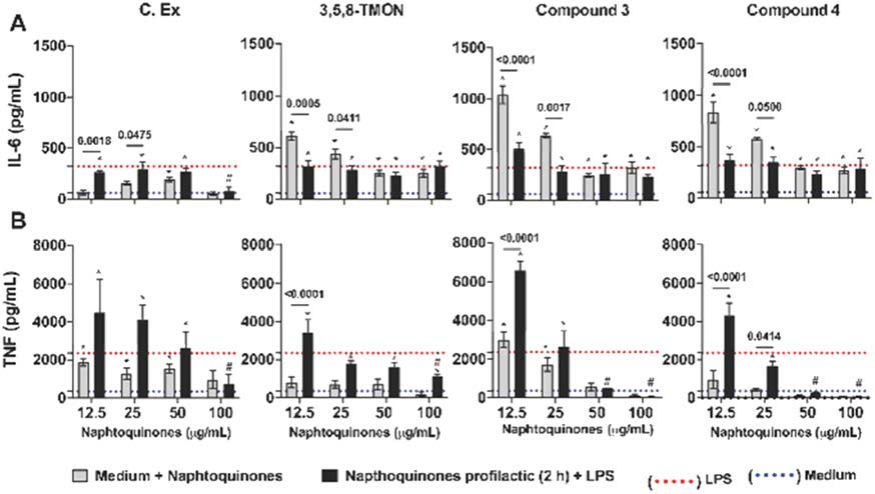
Naphthoquinone derivatives-treatment impact on cytokine production by human differentiate macrophages. (A) IL-6 and (B) TNF were measured by ELISA in the culture supernatant. The mean values of the positive control (macrophages treated with LPS 500 ng mL^−1^) are represented by the red dashed line and the negative control (medium) is represented by a blue dashed line in the graph. Data are expressed as mean ± SEM. Differences between groups are indicated by the *p*-values in the graphs and *p < 0.05 *vs.* medium (negative control) and #*p* < 0.05 *vs.* LPS (positive control).

While shikonin displays remarkable pharmacological activity, researchers have explored chemical modifications to its structure to enhance its pharmacological effects and mitigate potential toxicological concerns. The primary focus of these structural modification reactions has been on the side chain hydroxyl group. These modifications have resulted in the synthesis of various derivatives, including esters, oximes, glycosides, and many others.^24^ By enhancing selectivity, bioavailability, and reducing adverse effects, researchers seek to harness shikonin's anti-inflammatory properties more effectively in clinical settings. The ongoing exploration of shikonin derivatives underscores the dynamic nature of drug development, where structural modifications hold the promise of unlocking even greater efficacy and safety for addressing inflammatory conditions. Hence, we tested 3,5,8-TMON and its derivatives, using a human monocyte (THP-1) model exposed to the PMA phorbol ester, which differentiated to mature macrophages. During this differentiation process, NF-κB accumulates in the cytoplasm.^25^ In our study, we employed a prophylactic approach *in vitro* to treat LPS-stimulated macrophages with *Cordyceps* sp. crude extract (C. Ex), 3,5,8-TMON (2), compounds 3 and 4, at varying concentrations. An intriguing observation was made in the control group, where non-LPS-stimulated macrophages were treated with naphthoquinone compounds. Here, a significant increase in IL-6 production was observed as the concentration of the derivative compounds decreased, indicating an inverse correlation. This effect was not pronounced for the crude extract (Fig. 6A). In fact, treatment with crude extract (C. Ex) at a concentration of 100 μg mL^−1^ was the only treatment that demonstrated an inhibitory effect on IL-6 production by LPS-stimulated macrophages compared to treatment with derivative compounds (Fig. 6A).

Conversely, intriguing data emerged regarding TNF production (Fig. 5B). We observed that treatment with C. Ex and compound 3 resulted in the stimulation of TNF production, inversely proportional to the concentration of the compounds. Additionally, these treatments significantly enhanced the potential of LPS to induce TNF production in those differentiate macrophages. A similar effect of increased LPS-induced inflammatory potential was observed with the treatment of low concentrations of the 3,5,8-TMON and compound 4. However, at a concentration of 100 μg mL^−1^, all treatment compounds were capable of inhibiting TNF production by LPS-stimulated macrophages. Notably, compound 3 and 4 exhibited a potential anti-inflammatory effect compared to other treatments, with a dose of 50 μg mL^−1^ showing an inhibitory effect on TNF production (Fig. 6B). These results indicate that the compounds can function as agonists or antagonists in activated macrophages, depending on their concentration. This highlights the complex pharmacological interactions beyond a simple partial inhibition of NF-κB.

Our initial observation that the concentration of derivative compounds had an inverse correlation with IL-6 production in non-LPS-stimulated macrophages highlights the delicate balance of these compounds in regulating inflammation. It is important to note that while 3,5,8-TMON, compound 3, and 4 exhibited this inverse relationship with IL-6, the crude extract (C. Ex) did not follow the same pattern. This suggests that structural modifications may play a pivotal role in influencing the anti-inflammatory properties of these compounds. It should be noted that the precise mechanisms underlying this phenomenon warrant further investigation.

Additionally, our results demonstratedthat these compounds, particularly 3 and 4, exhibited significant anti-inflammatory potential at higher concentrations, by inhibiting TNF production. This dual behavior, acting as both pro-inflammatory and anti-inflammatory agents depending on concentration, highlights the intricate nature of pharmacological responses in activated macrophages. Moreover, the potential involvement of other transcription factors, such as AP-1, and innate immune receptors in these interactions should not be overlooked.^26^

### 3.4. Naphthoquinone-treatment 3,5,8-TMON influence on IL-6 production by SARS-CoV-2 infected cells

**Fig. 7:**
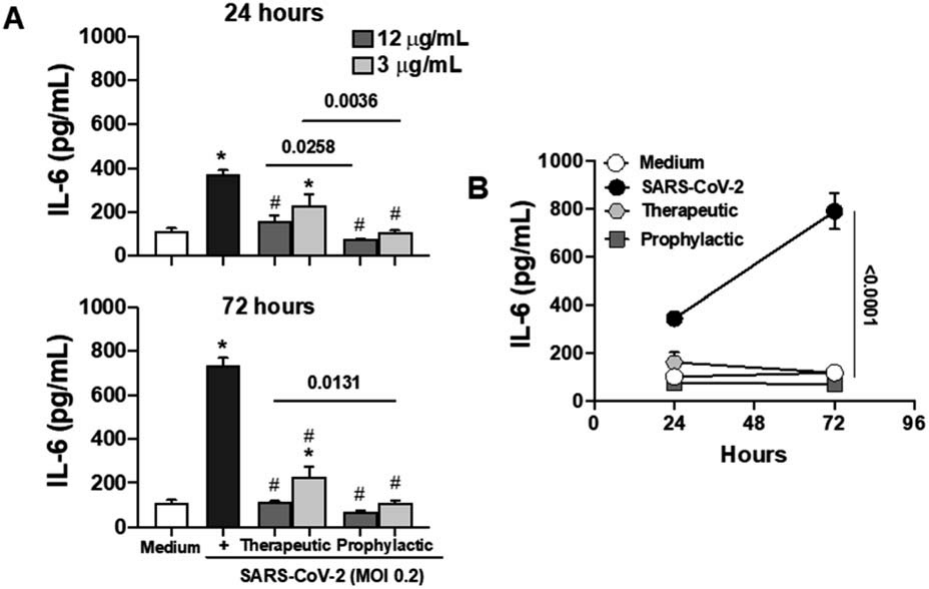
Treatment with naphthoquinone 3,5,8-TMON induced inhibition of IL-6 secretion in airway epithelial cells infected with SARS-CoV-2. Calu-3 cells were infected with SARS-CoV-2 at multiplicity of infection (MOI) 0.2. (A) IL-6 concentration was measured in supernatants derived from non-infected and SARS-CoV-2 infected cells at 24 and 72 hours p.i. (B) Longitudinal analysis of time-course IL-6 production in infected cells and naphthoquinones-treated. Data represent the mean ± SD of at least two independent experiments performed in triplicate. *p < 0.05 *vs.* medium and #*p* < 0.05 *vs.* SAR-CoV-2 infection. The bars represent the *p*-values when compared to the different treatments approach (therapeutic *vs.* prophylactic).

Considering the pivotal role of IL-6 in immune dysregulation during infections,^27^ and the demonstrated ability of naphthoquinones to reduce cytokine production in LPS-stimulated macrophages, we sought to evaluate their anti-inflammatory effects in epithelial Calu-3 cells infected with SARS-CoV-2. Our approach involved both prophylactic and therapeutic treatments with naphthoquinones. Remarkably, our findings revealed a significant reduction in IL-6 secretion during the 24 and 72 hours time courses of viral infection *in vitro*, following treatment with 3,5-8TMON. This effect was consistently observed at concentrations of 3.12 and 12.5 μg mL^−1^ for both treatment approaches (Fig. 7A). In a longitudinal analysis, we further noted that IL-6 production increased from 24 to 72 hours after infection, yet the inhibitory effect of naphthoquinones persisted, effectively controlling this cytokine production throughout this period (see Fig. 7B). Furthermore, our investigation extended to the evaluation of the viability of Calu-3 cells after SARS-CoV-2 infection. Remarkably, at the concentrations used, we did not observe any discernible cell death before or after infection.

These results suggest a promising potential for naphthoquinones in mitigating the dysregulated immune responses associated with viral infections, particularly SARS-CoV-2. The ability to modulate IL-6 production, a key contributor to the cytokine storm observed in severe COVID-19 cases,^28^ underscores the importance of further investigations into the therapeutic utility of naphthoquinones in the context of viral infections. These findings contribute to our growing understanding of the multifaceted roles of natural compounds in modulating immune responses and warrant further exploration in preclinical and clinical studies.

Cytokines play a central role in immune responses, particularly in macrophages activation and subsequent synthesis of pro-inflammatory cytokines, such as IL-1B and TNF.^29^ Notably, recent research by Liu *et al.* has demonstrated the potential of a 1,4-naphthoquinone derivative, specifically 6-[1-(acetyloxy)ethyl]-5-hydroxy-2,7-dimethoxy-1,4-naphthalenedione, to attenuate pro-inflammatory cytokines, specifically IL-1β, IL-6, and TNF in LPS-stimulated murine macrophages (RAW 264.7 cells).^30^ In our present study, using different cell types including differentiated THP-1 macrophage and epithelial Calu-3 cells, we expanded the scope of investigation to explore the immunomodulatory effects of novel naphthoquinone derivatives on inflammation *in vitro*. Regulation of LPS-induced TNF expression is known to involve the NF-κB pathway, mediated by activation of the PI3K/AKT pathway, with reliance on the IRAK1 enzyme.^31^ Our findings reveal the remarkable efficacy of these newly synthesized naphthoquinone derivatives in suppressing critical pro-inflammatory cytokines, such as TNF-α and IL-6, as well as nitric oxide production (NO). The ability of these derivatives to target key signaling pathways associated with inflammation, such as the NF-κB and PI3K/AKT pathways, highlights their potential as potent immunomodulators. Moreover, their capacity to inhibit NO production underscores their role in dampening the oxidative stress often associated with exacerbated inflammation. This diversity suggests that these naphthoquinone derivatives may have wide-ranging applications in modulating immune responses in different contexts, including infections, autoimmune disorders, and chronic inflammatory conditions.

## 4 Conclusions

In summary, the strategy employed for the production and extraction of naphthoquinone metabolites from the fungus *Cordyceps* sp. has proven to be robust and effective, providing substrates for biological evaluation and for structural derivatization studies. Acidification of the reaction medium in carbonyl substitution reactions for 3,5,8-TMON enhanced reactivity, resulting in increased formation of final products, as demonstrated in the reactions between 3,5,8-TMON and phenyl hydrazine. Using NMR and mass spectrometry techniques, we confirmed the isolation of new compounds with significant potential as novel naphthoquinone-based drug candidates bearing the dihydropyrazole ring and the hydrazone group. Our study has revealed the promising anti-inflammatory properties of these 1,4-naphthoquinone derivatives, highlighting their potential as preventive agents against inflammation-related disorders. The nuanced impact of dose and timing on their efficacy opens avenues for future research aimed at optimizing its therapeutic application. These compounds exhibit the capacity to act as both agonists and antagonists, depending on their concentrations and the specific inflammatory milieu. The structural modifications made to these compounds offer exciting avenues for drug development, with potential applications in both preventing and treating inflammatory conditions. However, further research into the underlying mechanisms is warranted to fully harness their therapeutic potential and address their complexities. This work underscores the dynamic nature of pharmacological research in the pursuit of effective anti-inflammatory agents from natural sources. The synthesis and biological evaluation of novel 3,5,8-TMON analogues are currently being investigated in our laboratories.

## Author contributions

L. A. B. M. and G. C. C. conceptualized the project. J. N. M. cultivated the fungi and J. N. M. and V. T. M. developed the extraction methodology. Synthetic methodologies were developed by V. T. M. and F. J. C. J. N. M. and V. T. M. developed the analytical methods for the chemical compounds. V. T. M., J. N. M., L. A. B. M., G. C. C. analysed the spectral data. B. B. F. worked on biological assays in cells Raw Blue™ macrophages under supervision of P. D. M. P. V. SN. worked on biological assays in cells THP-1 and P. V. SN. and T. F. C. FS worked on biological assays in cells Calu-3 and SARS-CoV-2 infection assays under supervision of C. A. S. and V. L. D. B. Biological data were analysed by P. V. SN. and T. F. C. FS., C. A. S. was responsible for the formal analysis. L. A. B. M. and G. C. C. supervised the research. L. A. B. M. and G. C. C. were responsible for funding acquisition and resources. L. A. B. M. and G. C. C. were responsible for validation. V. T. M., G. C. C. and L. A. B. M. wrote the original draft and edited the text. P. V. SN., C. A. S., V. L. D. B. and P. D. M. edited the text.

## Conflicts of interest

There are no conflicts to declare.

## Supplementary Material

RA-014-D3RA08173G-s001
