# Supplementary material for: Naphthoquinone derivatives as potential immunomodulators: prospective for COVID-19 treatment
Source: RSC Adv. 2024 Feb 21;14(10):6532–41. doi: 10.1039/d3ra08173g (PMC10880745; doi:10.1039/d3ra08173g)
Supplement: RA-014-D3RA08173G-s001 [file RA-014-D3RA08173G-s001.pdf]

Electronic Supplementary Material (ESI) for RSC Advances.
This journal is © The Royal Society of Chemistry 2024

Supplementary Information for:

Naphthoquinone Derivatives as Potential Immunomodulators: Prospective for COVID-19 Treatment

Vitor Tassara Moraes[a], Franco Jazon Caires[a], Pedro V. da Silva-Neto[b], Jacqueline Nakau Mendonça[a], Thais F. C. Fraga-Silva[c,d], Bianca Bueno Fontanezi[e], Priscyla Daniely Marcato[e], Vania Luiza Deperon Bonato[d], Carlos Arterio Sorgi[b], Luiz Alberto Beraldo Moraes[b], Giuliano Cesar Clososki[a]*

[a] Departamento de Ciências Biomoleculares, Faculdade de Ciências Farmacêuticas de Ribeirão Preto, Universidade de São Paulo-USP, Ribeirao Preto 14040-903, SP, Brazil.
[b] Departamento de Química, Faculdade de Filosofia, Ciências e Letras de Ribeirão Preto-FFCLRP, Universidade de São Paulo-USP, Ribeirao Preto 14040-901, SP, Brazil.
[c] Instituto de Ciências Biológicas e da Saúde, Universidade Federal de Alagoas- UFAL, Maceió 57072-900, AL, Brazil
[d] Departamento de Bioquímica e Imunologia, Faculdade de Medicina de Ribeirão Preto-FMRP, Universidade de São Paulo-USP, Ribeirao Preto 14040-900, SP, Brazil
[e] Departamento de Ciências Farmacêuticas, Faculdade de Ciências Farmacêuticas de Ribeirão Preto, Universidade de São Paulo-USP, Ribeirão Preto 14040-903

1. Experimental spectra

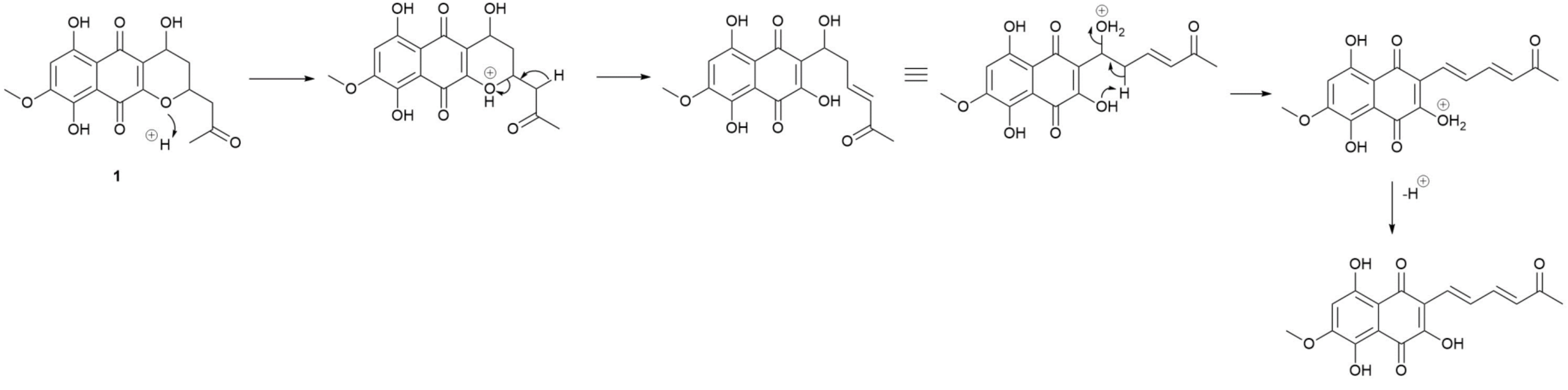

1

2

Figure S1. Plausible mechanism for the conversion of Erythrostominone (1) into 3,5,8-TMON (2).

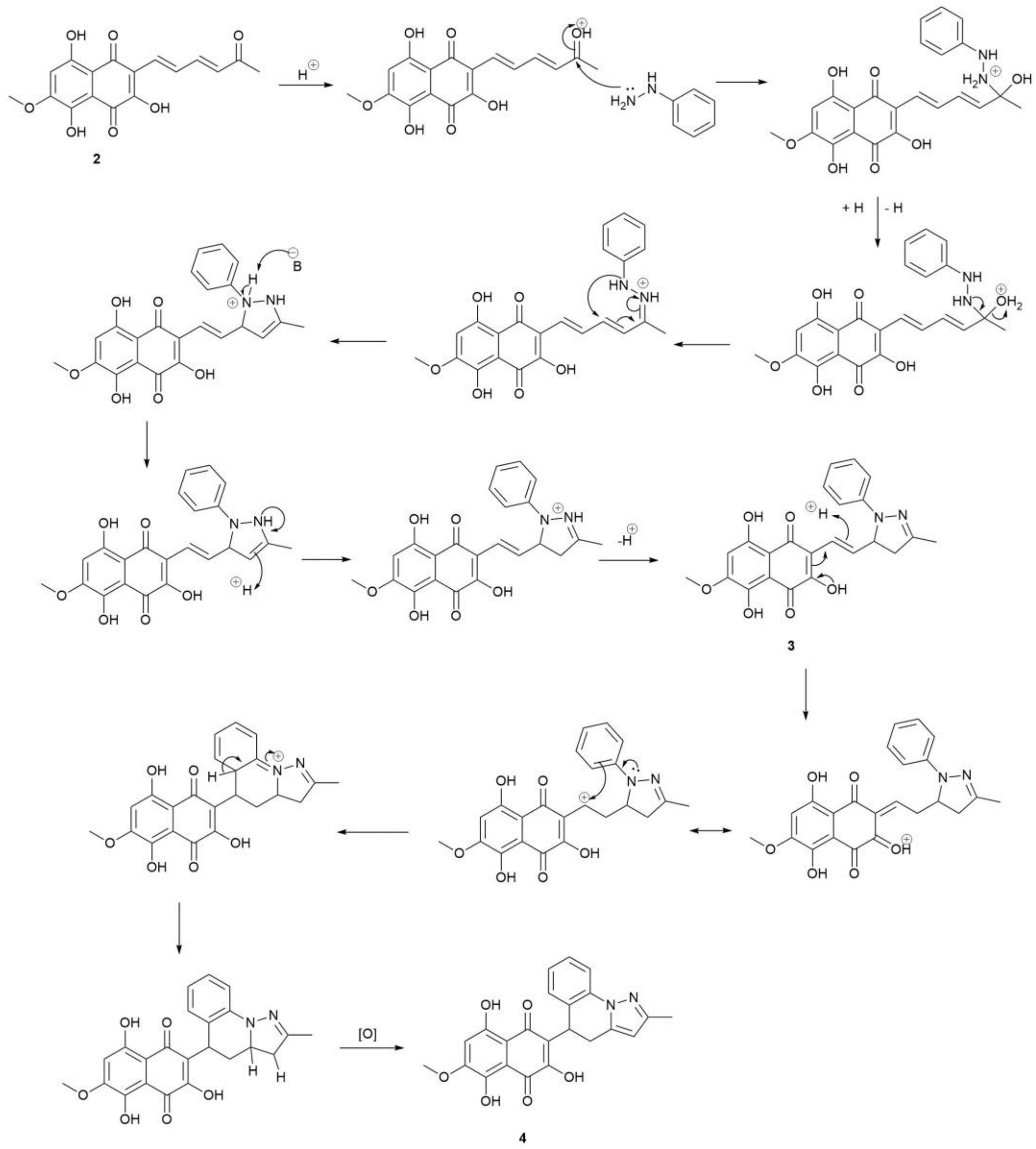

Figure S1. Plausible mechanism for the synthesis of 3 and 4.

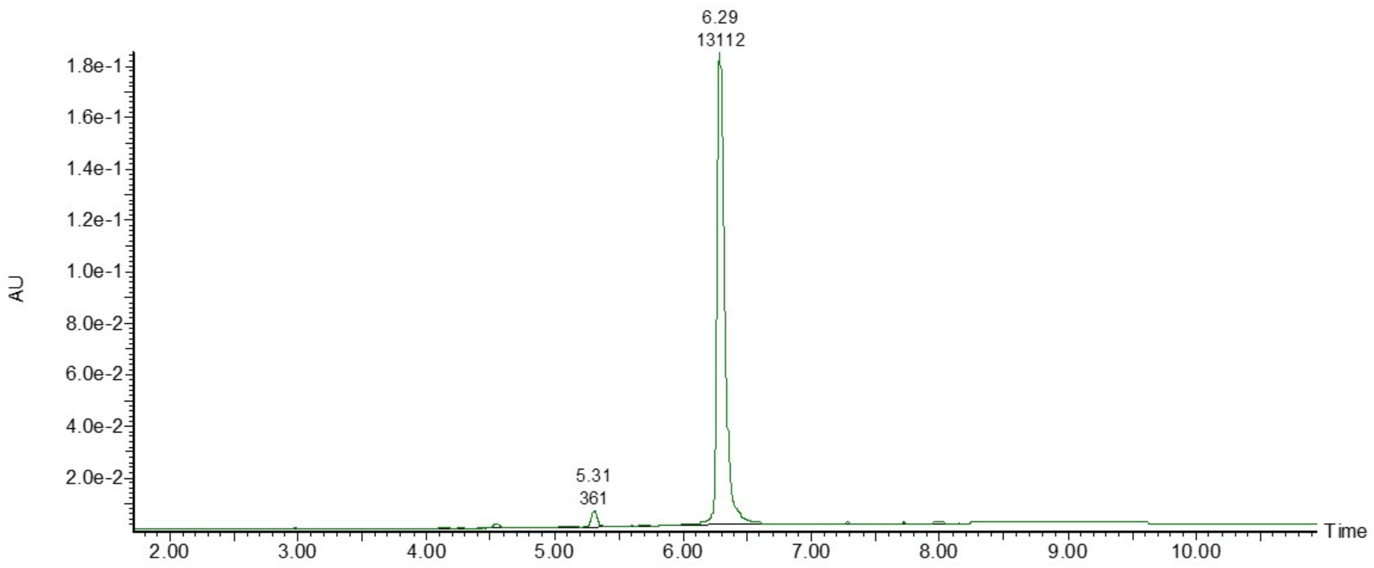

Figure S2. 3,5,8-TMON (2) UPLC-DAD chromatogram. Relative peak purity (97.3%).

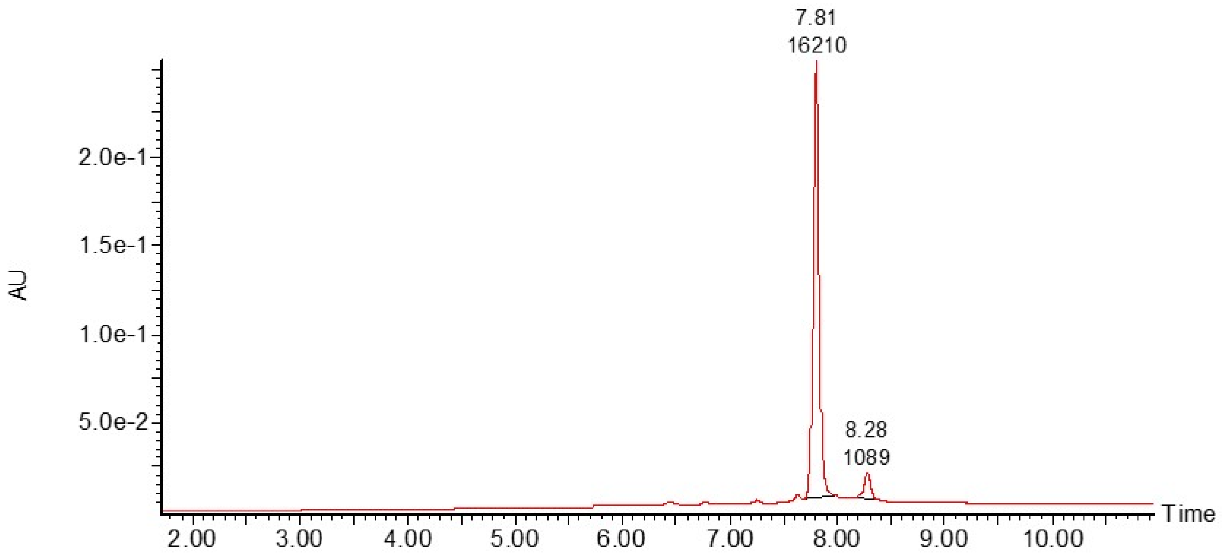

Figure S3. Compound 3 UPLC-DAD chromatogram. Relative peak purity (93.7%)

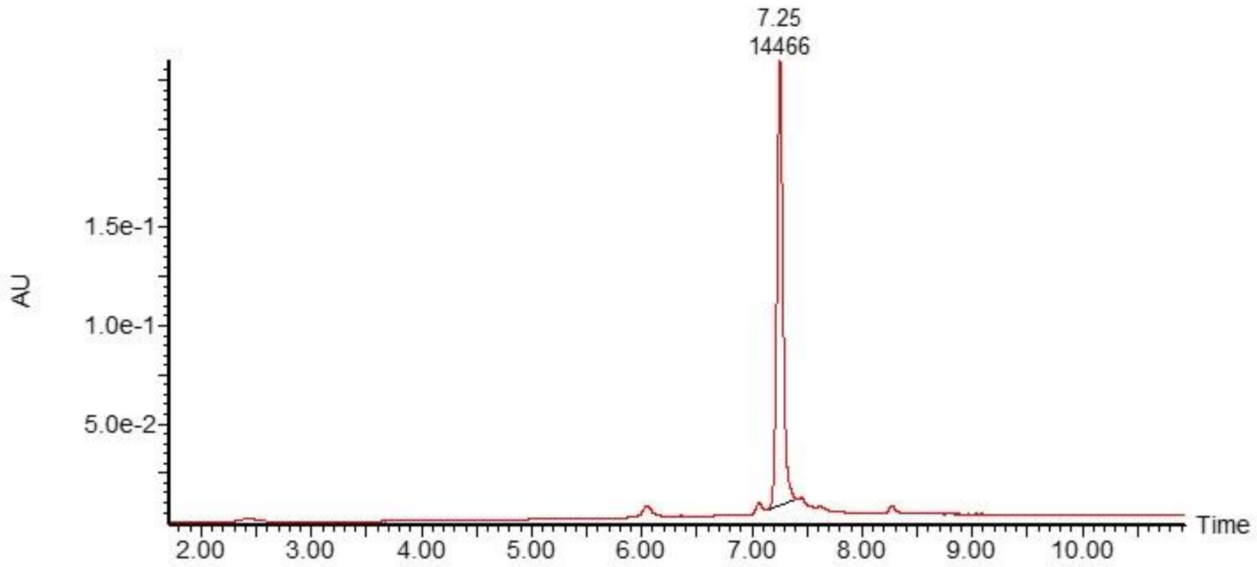

Figure S4. Compound 4 UPLC-DAD. Relative peak purity (~100%)

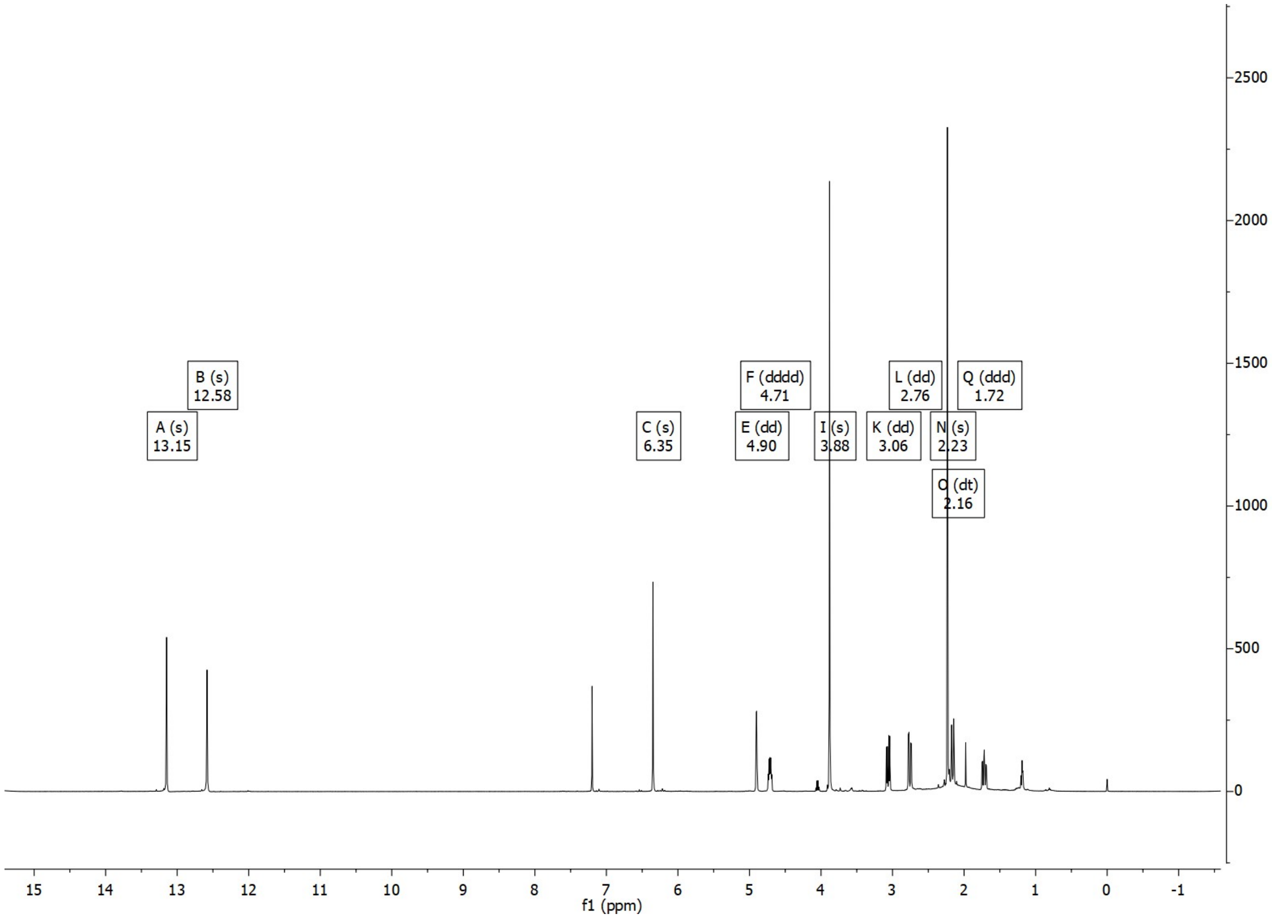

Figure S5. [1]**H NMR spectra of Erythrostominone in** $CdCl_3$**, 500 MHz.**

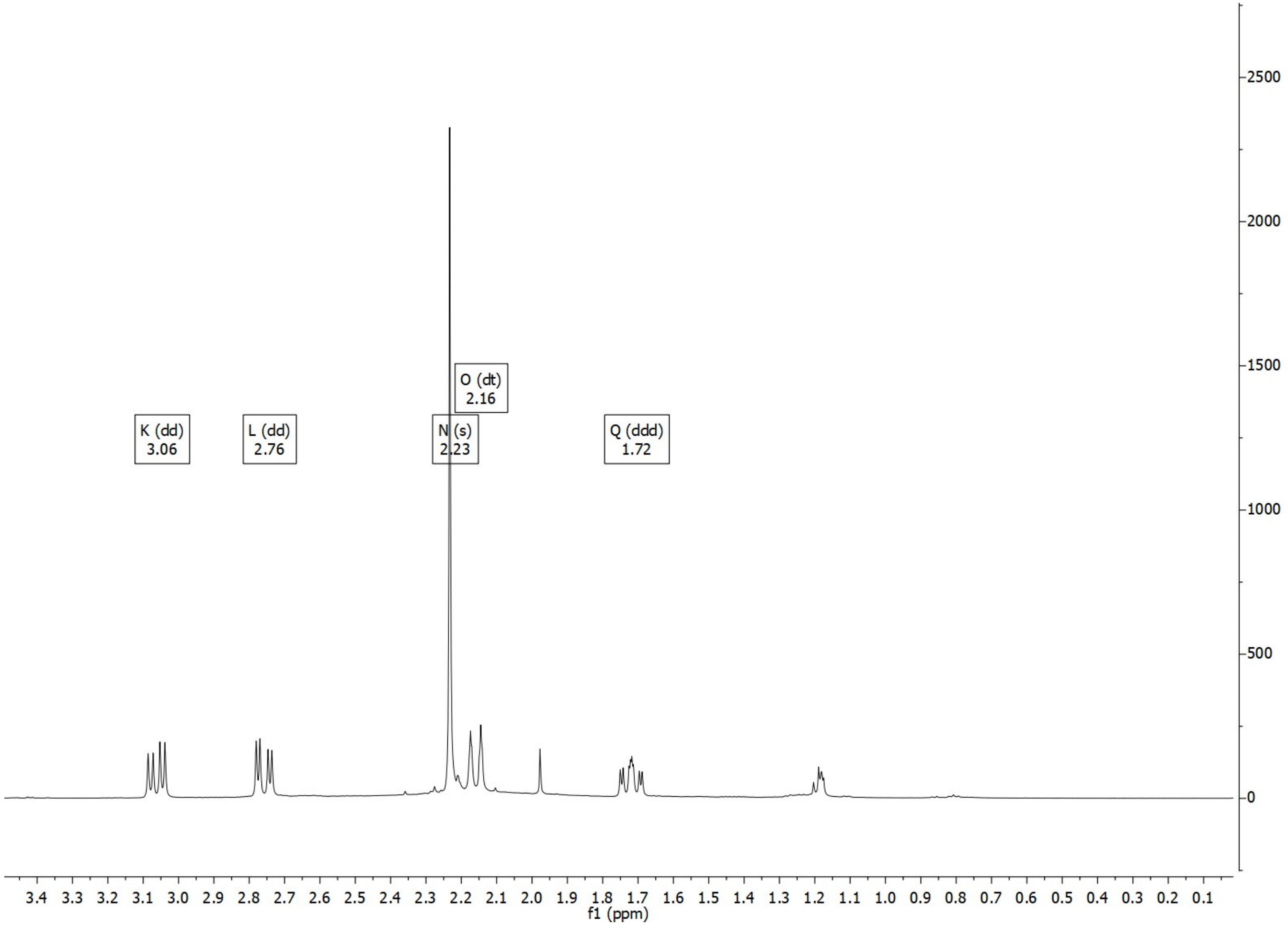

Figure S6. ^{1}H NMR spectra of Erythrostominone from 0 to 3.5 ppm in $CDCl_3$, 500 MHz.

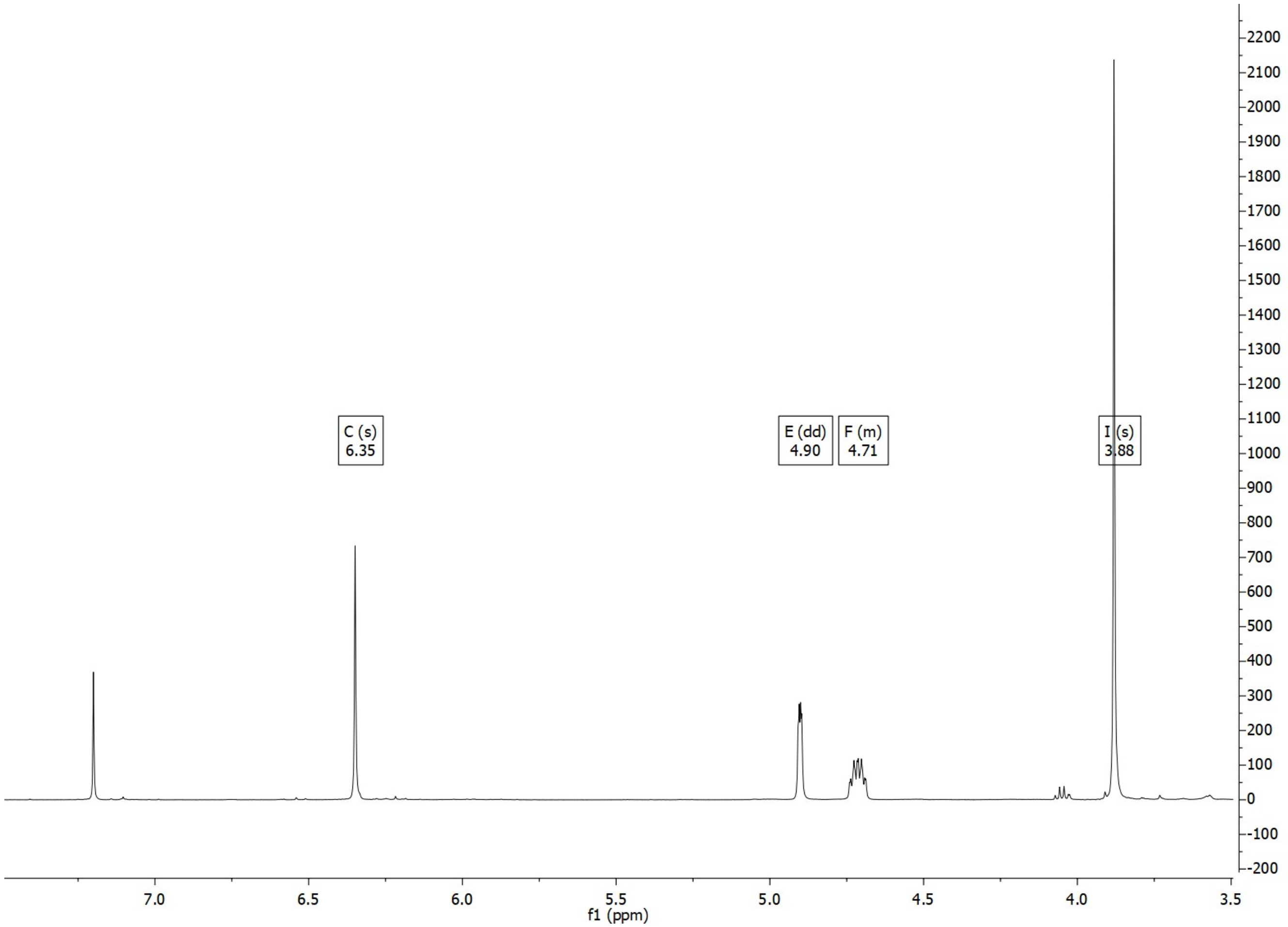

Figure S7. ^{1}H NMR spectra of Erythrostominone from 3.5 to 7.5 ppm in $CDCl_3$, 500 MHz.

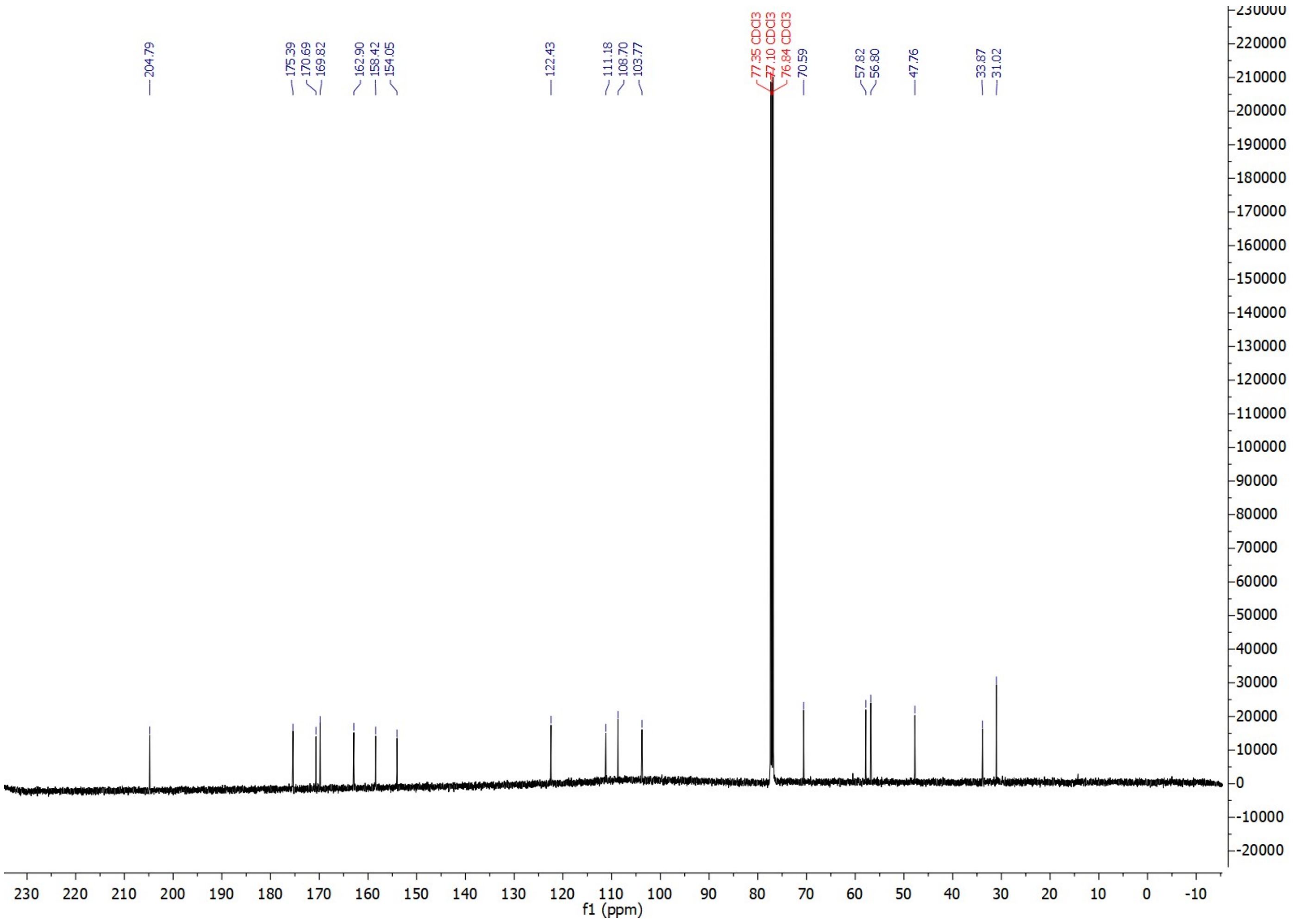

Figure S8. **^{13}C NMR spectra of Erythrostominone in $CDCl_3$, 125 MHz.**

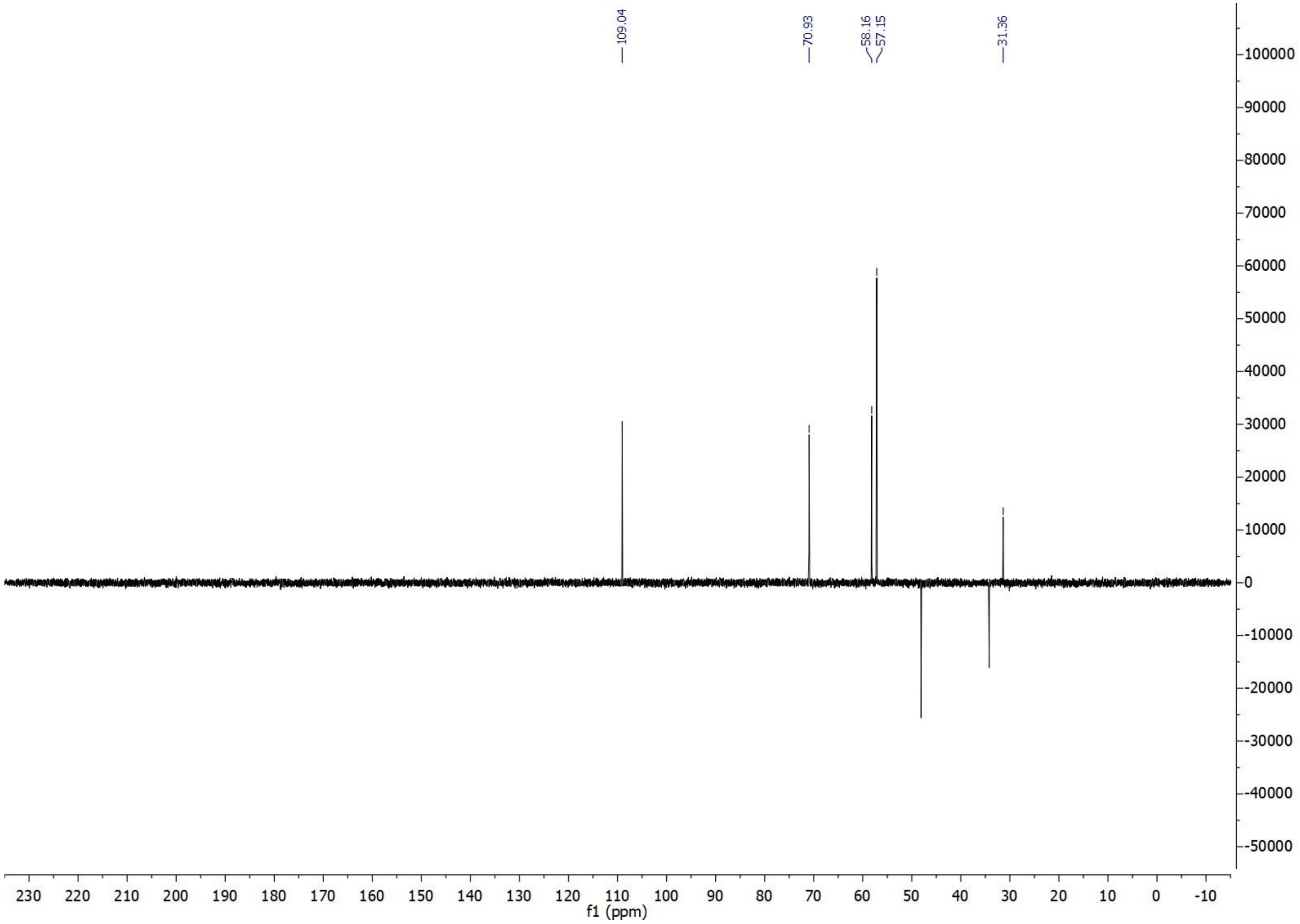

Figure S9. DEPT-136 NMR spectra of Erythrostominone in $CDCl_3$.

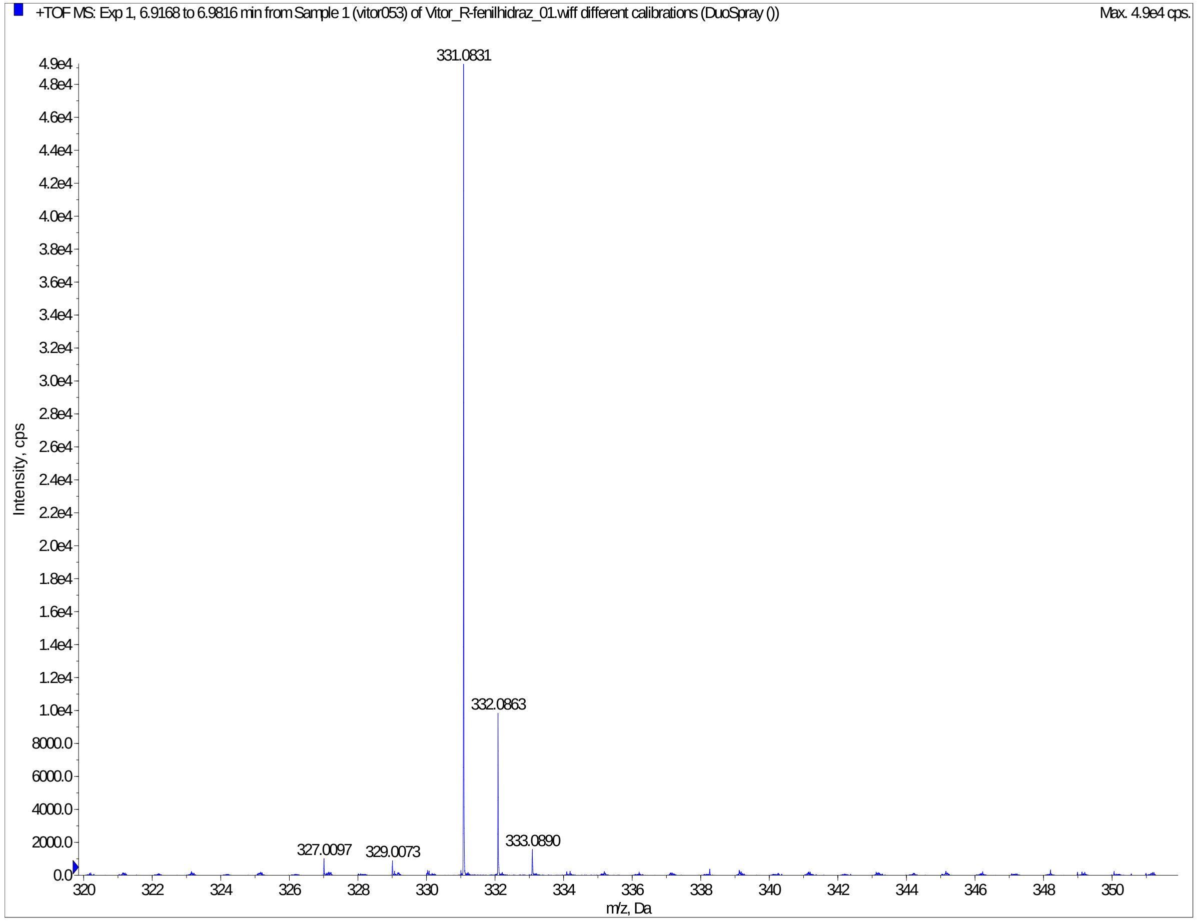

Figure S11. HRMS ESI+ spectra obtained in positive mode for 3,5,8-TMON (2). The molecular formula $C_{17}H_{14}O_7$ corresponds to the exact calculated mass of 330.0740 u for the deprotonated molecule, with a 3.9385 ppm error compared to the spectra.

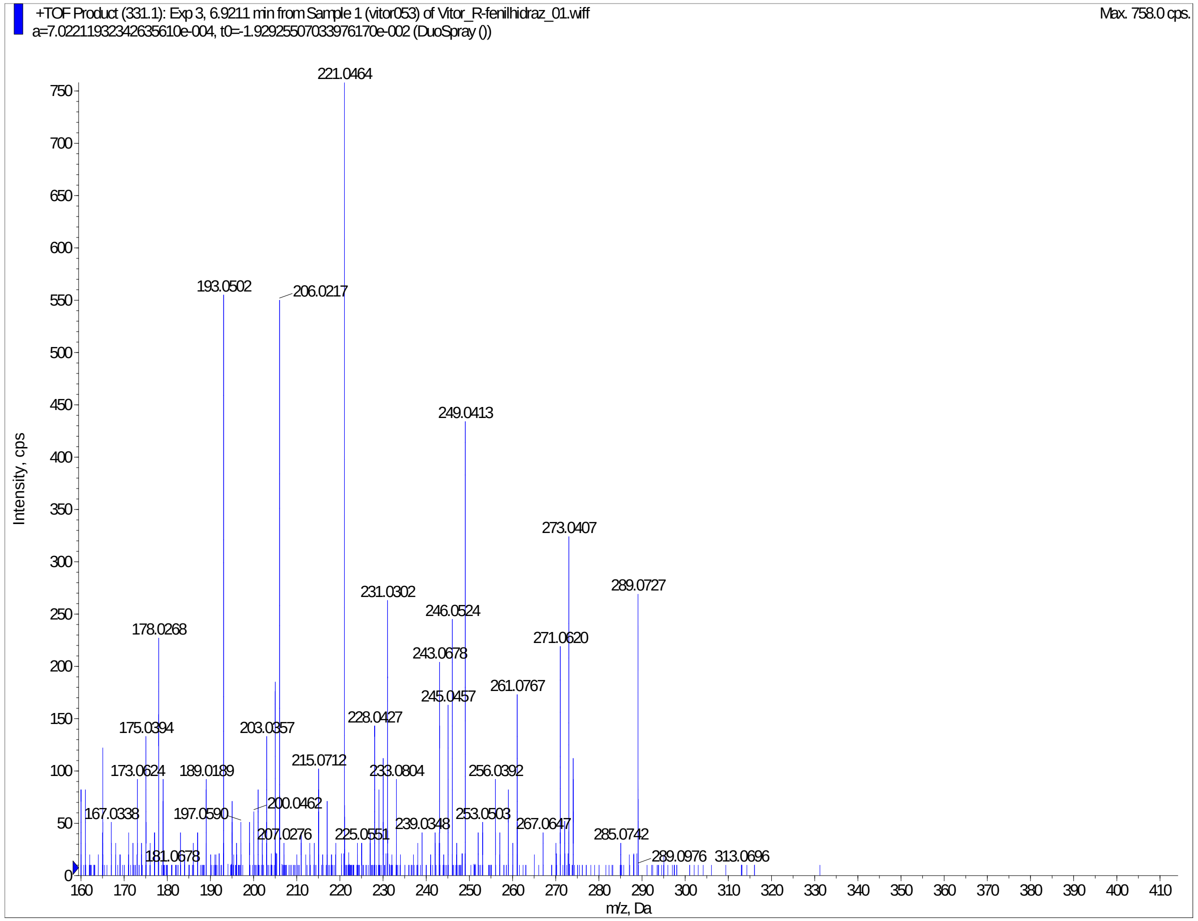

Figure S12. ESI+ CID spectra (30eV, N_2) of 3,5,8-TMON (2).

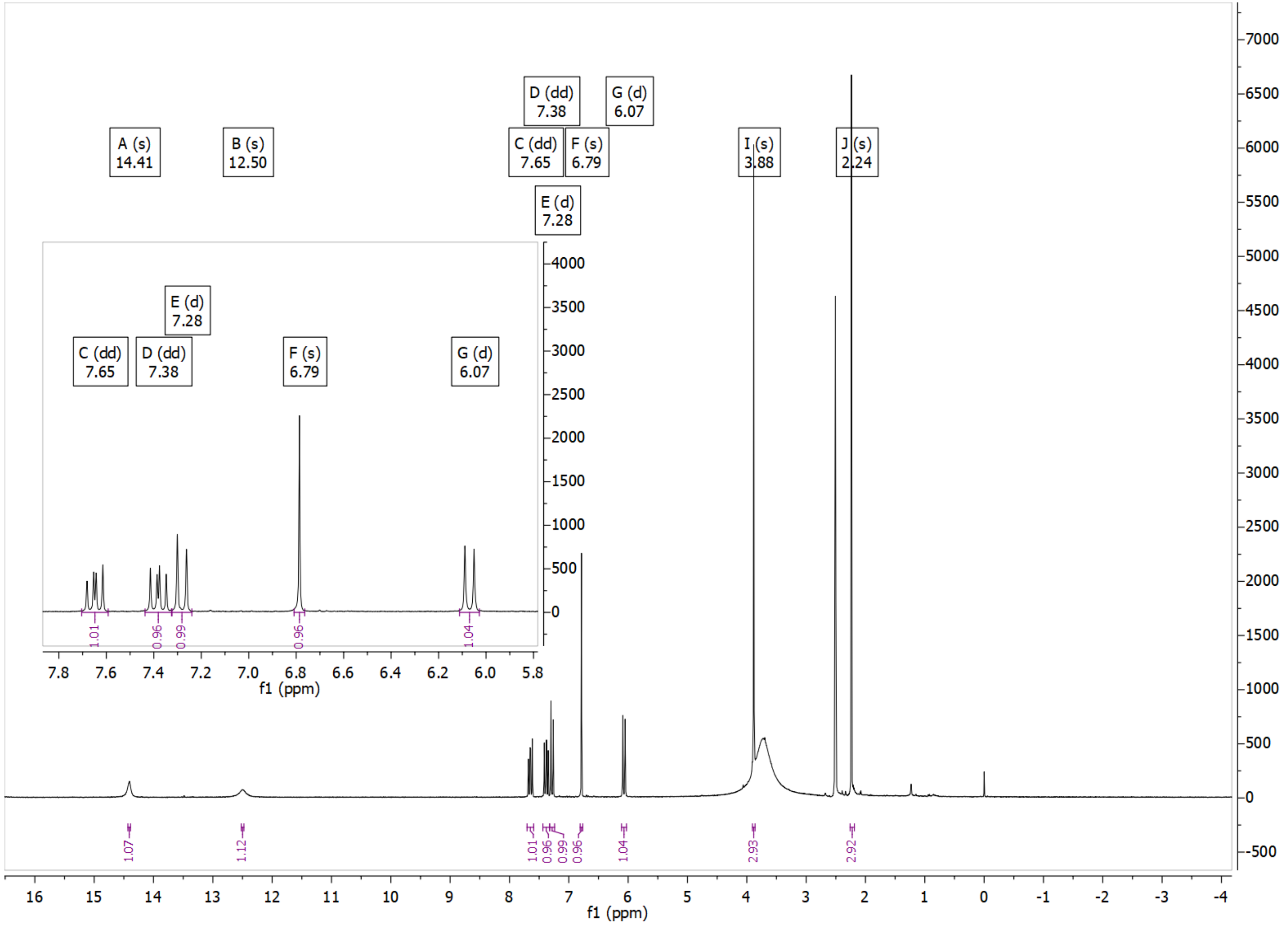

Figure S12. **^{1}H NMR spectra of 3,5,8-TMON in DMSO-d_6, 400 MHz.**

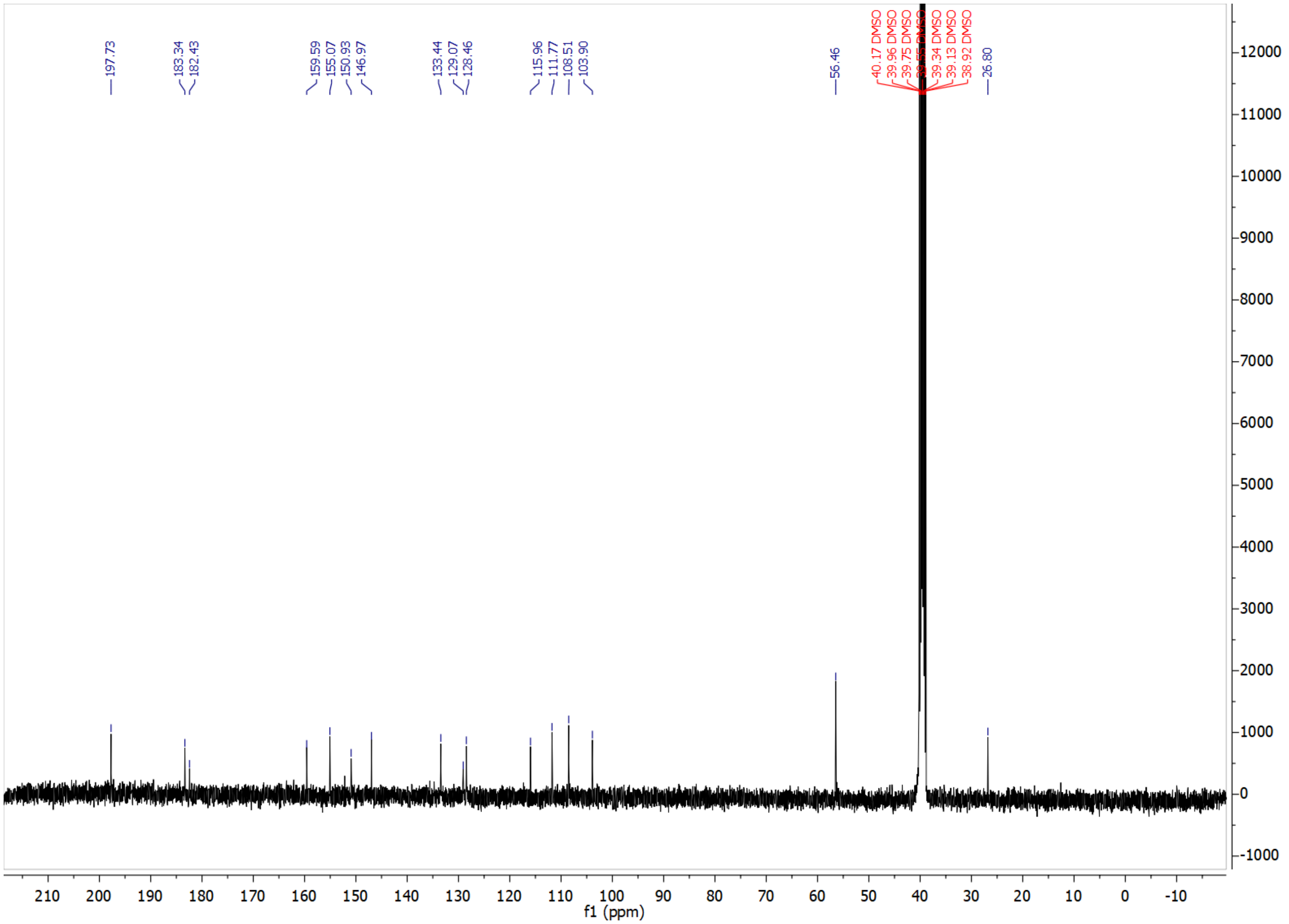

Figure S13. ^{13}C **NMR spectra of 3,5,8-TMON in DMSO-d_6, 100 MHz**

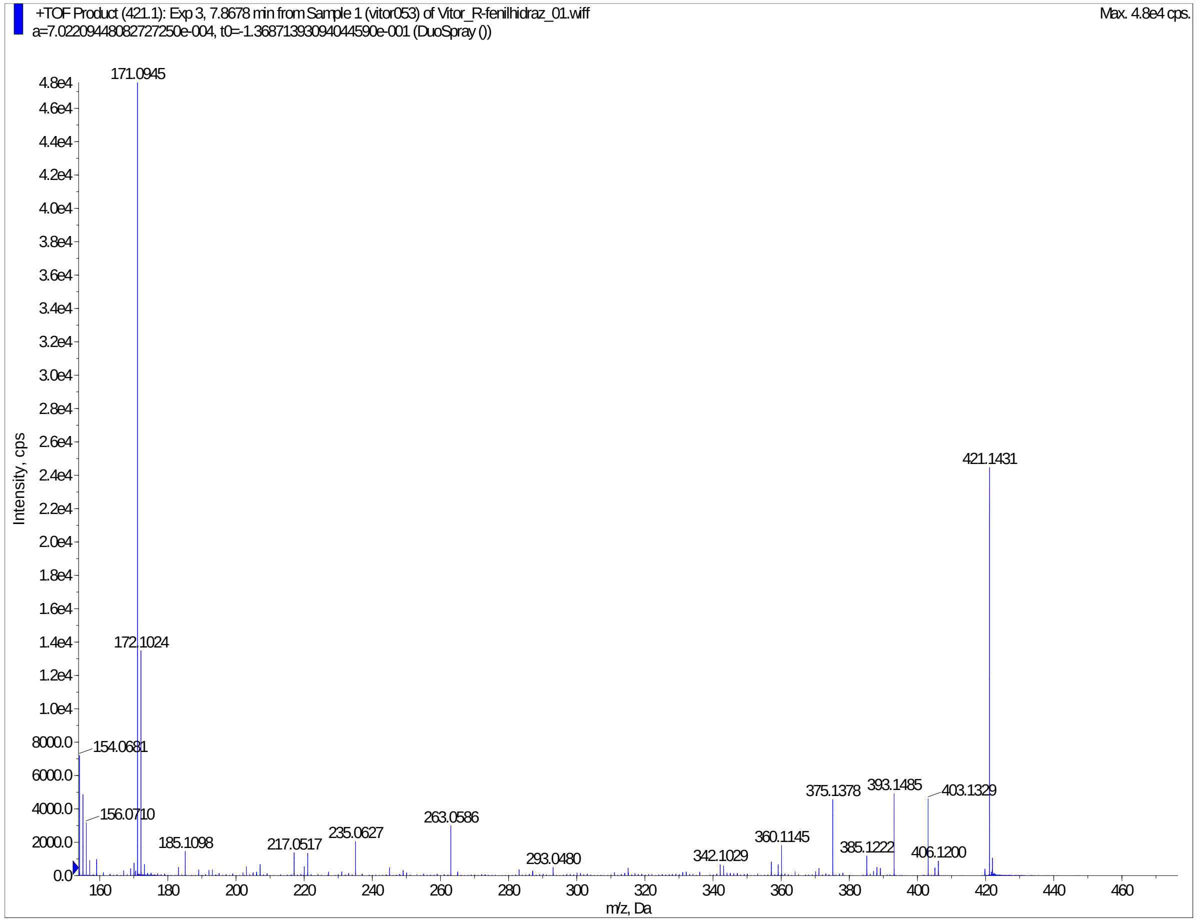

Figure S14. ESI+ CID spectra (30eV, N_2) of 3.

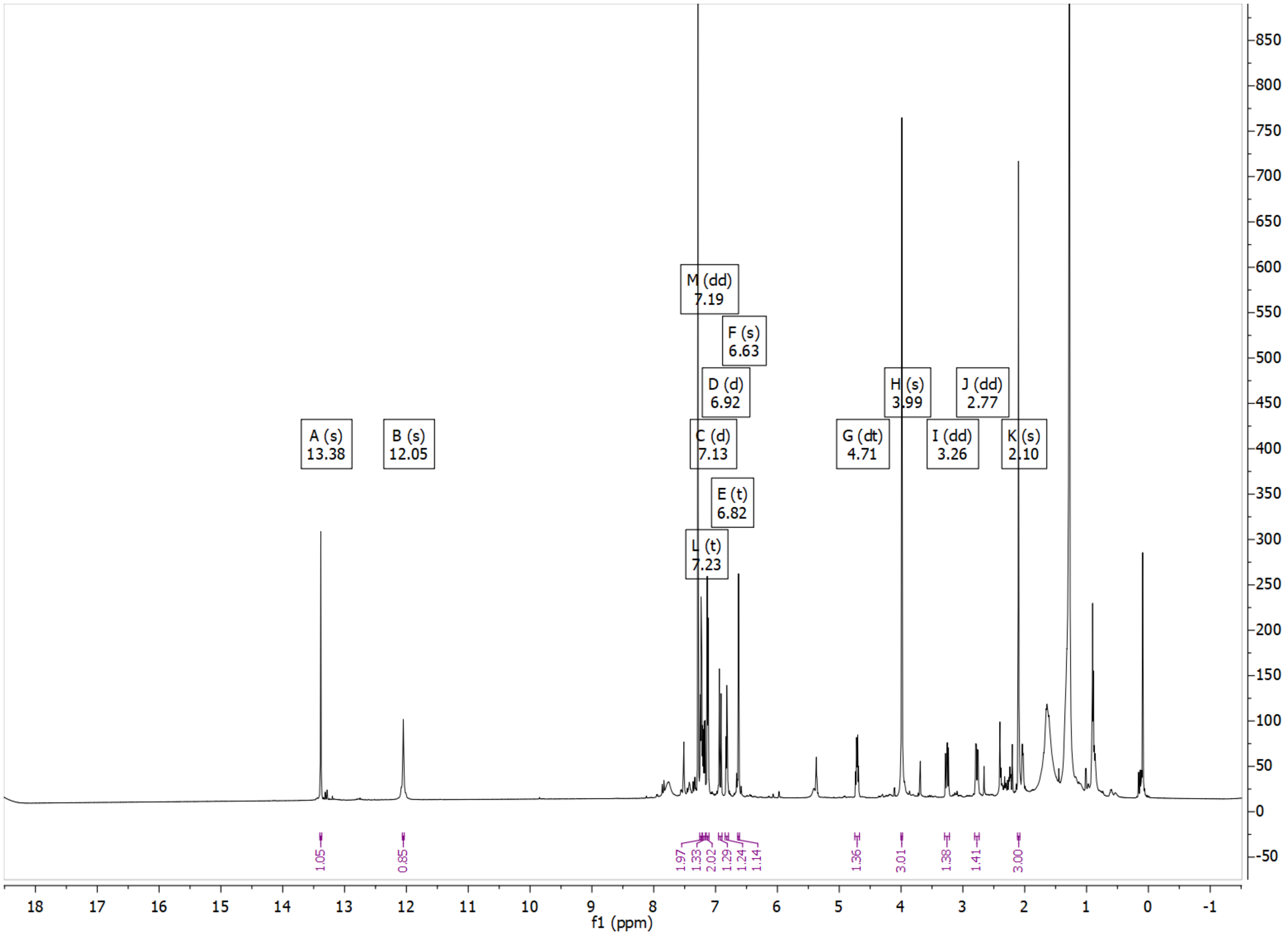

Figure S15. **^{1}H NMR spectra of 3 in $CDCl_3$, 600 MHz.**

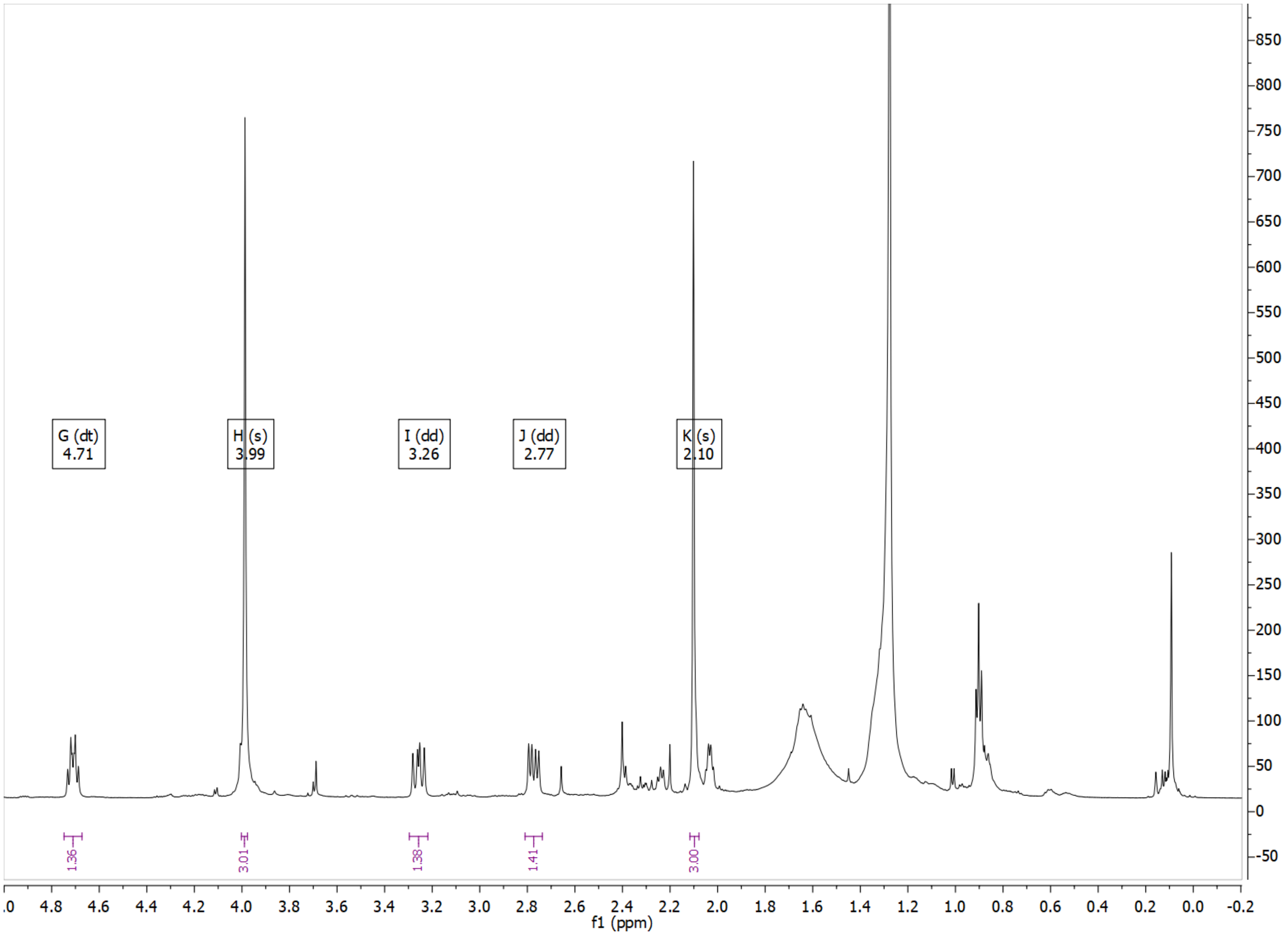

Figure S16. **^{1}H NMR spectra of 3 from 0 to 5 ppm in $CDCl_3$, 600 MHz.**

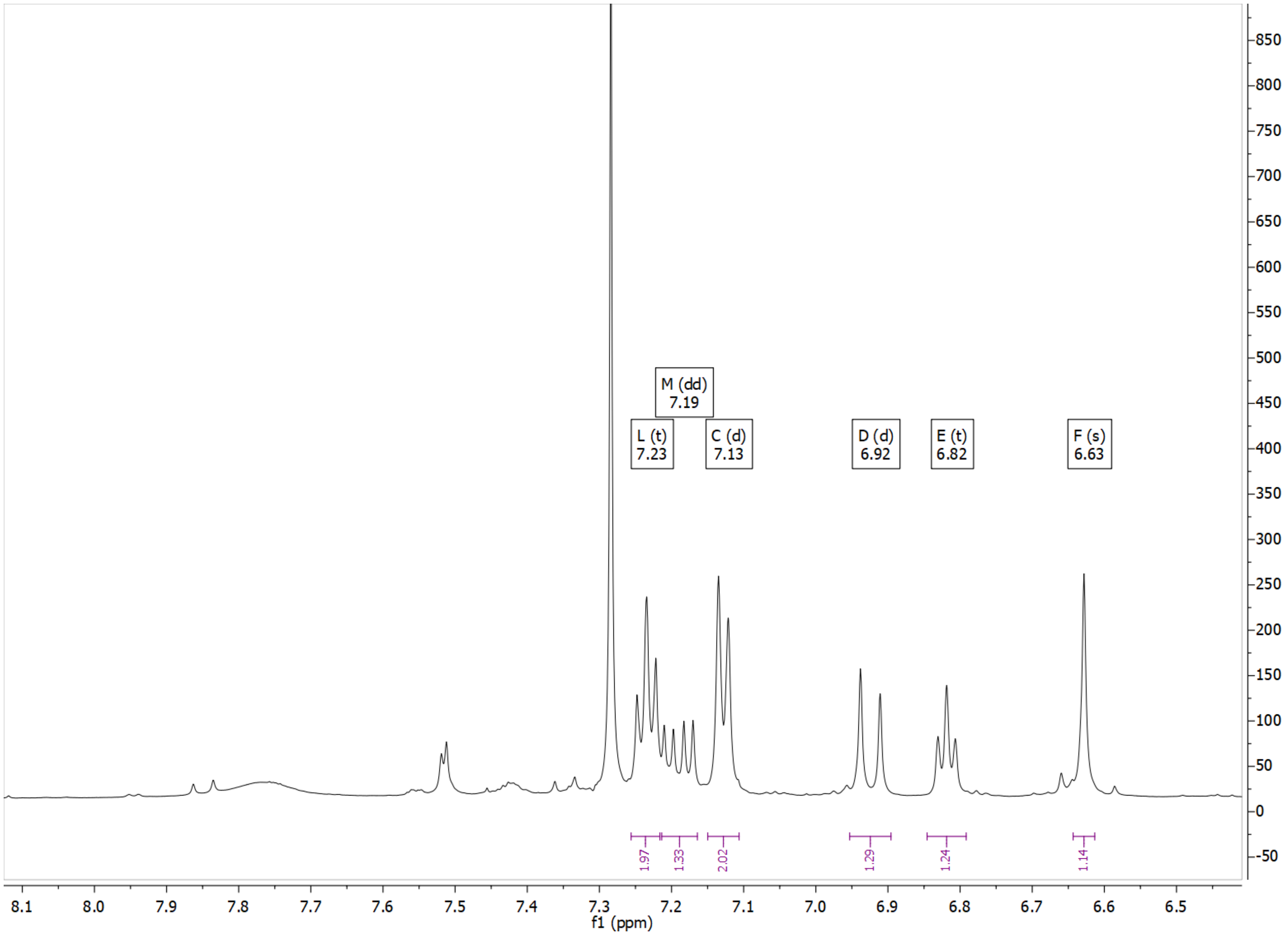

Figure S17. ^{1}H NMR spectra of 3 from 6.5 to 8.0 ppm in $CDCl_3$, 600 MHz.

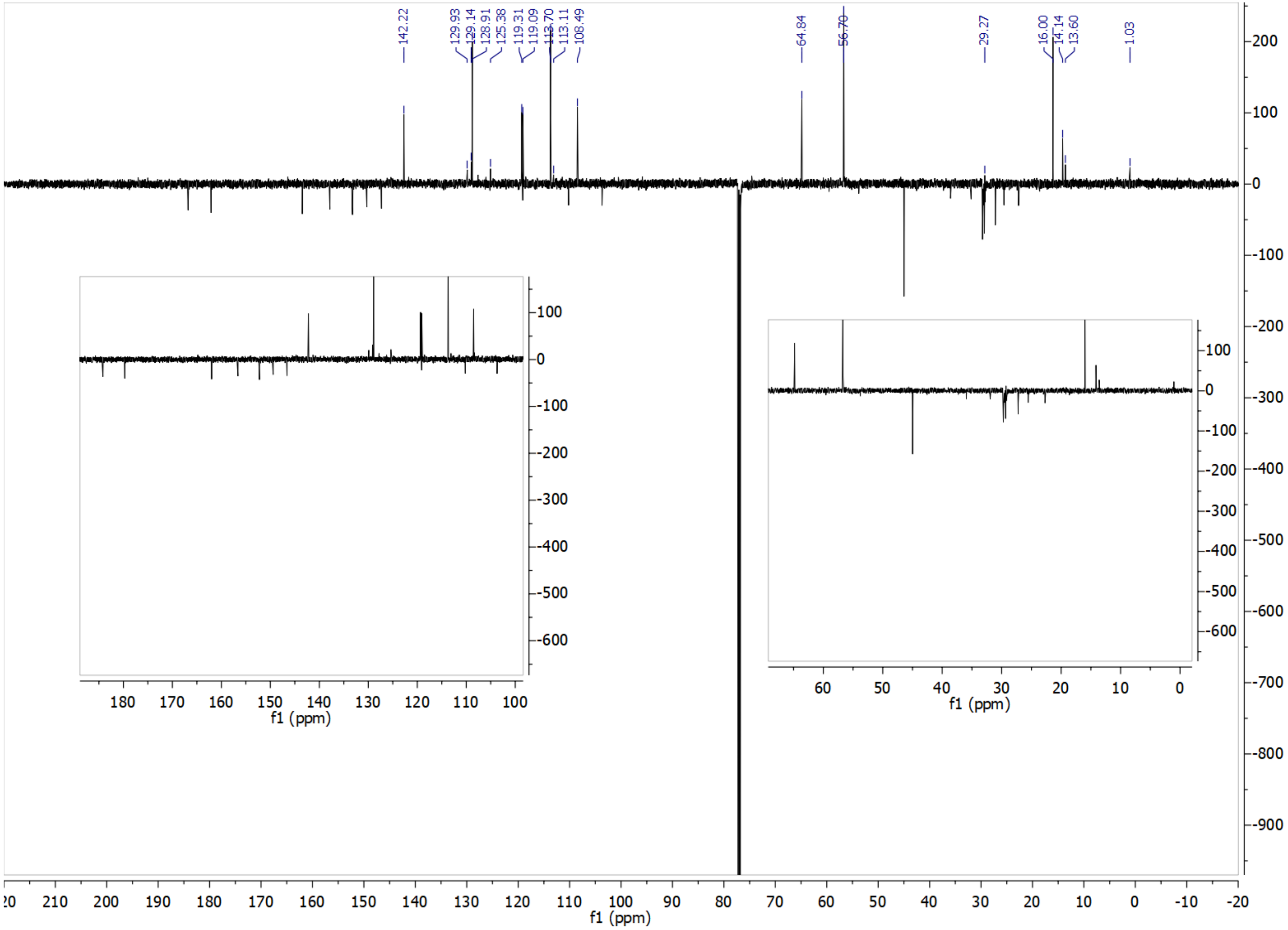

Figure S18. ^{13}C **APT NMR spectra of 3 in** $CDCl_3$**, 150 MHz.**

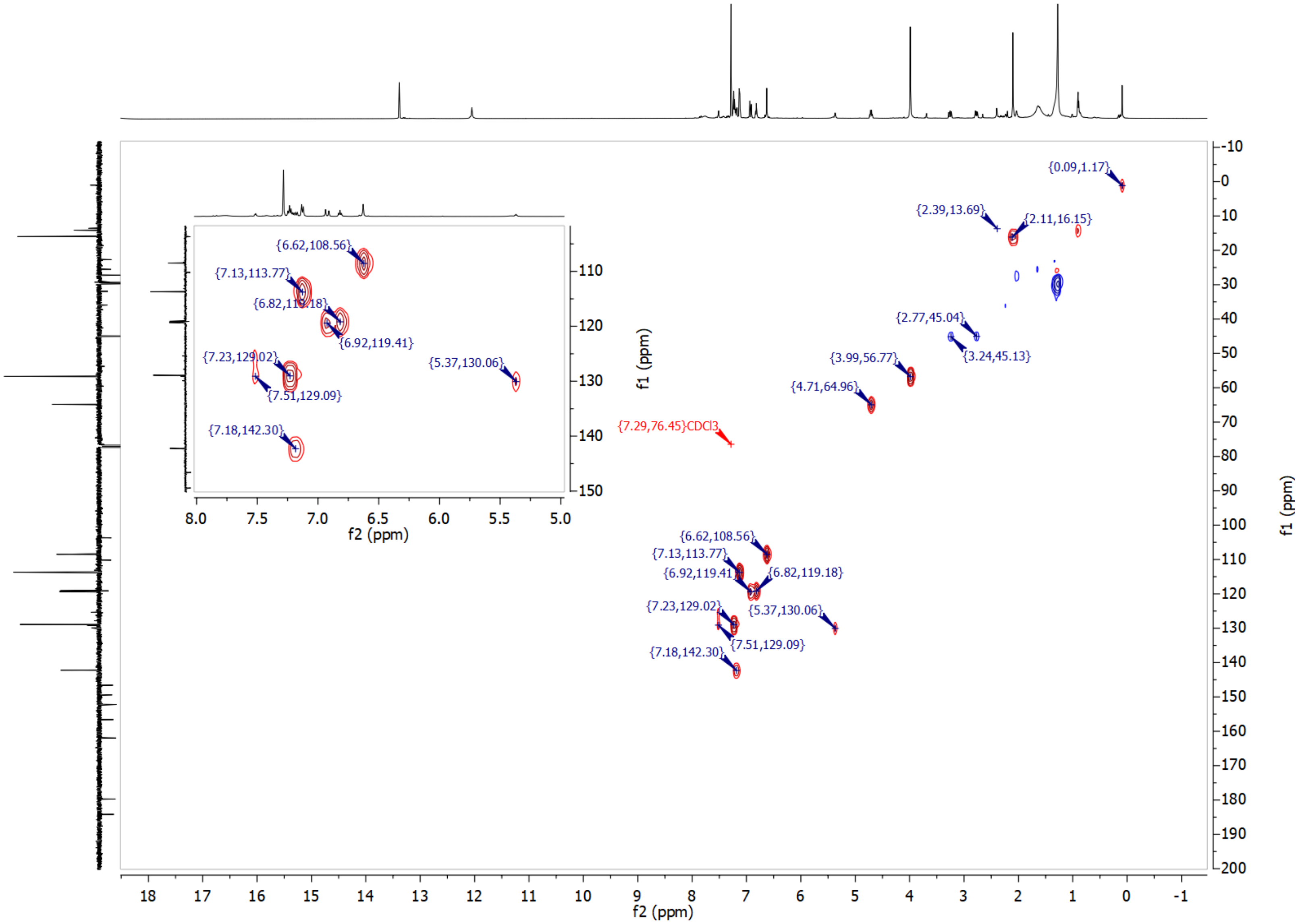

Figure S20. HMQC map for 3 in $CDCl_3$.

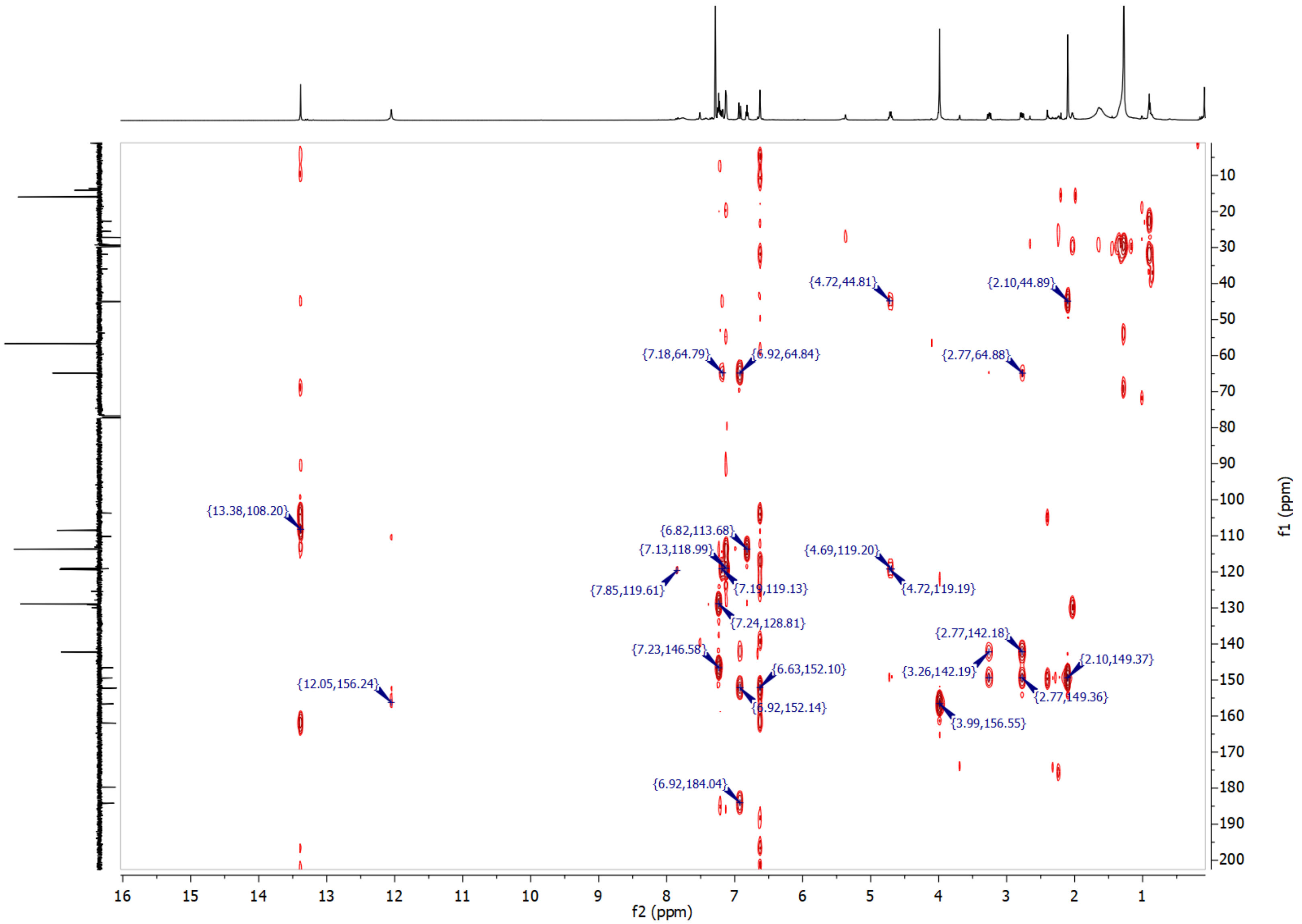

Figure S29. HMBC map for 3 in $CDCl_3$.

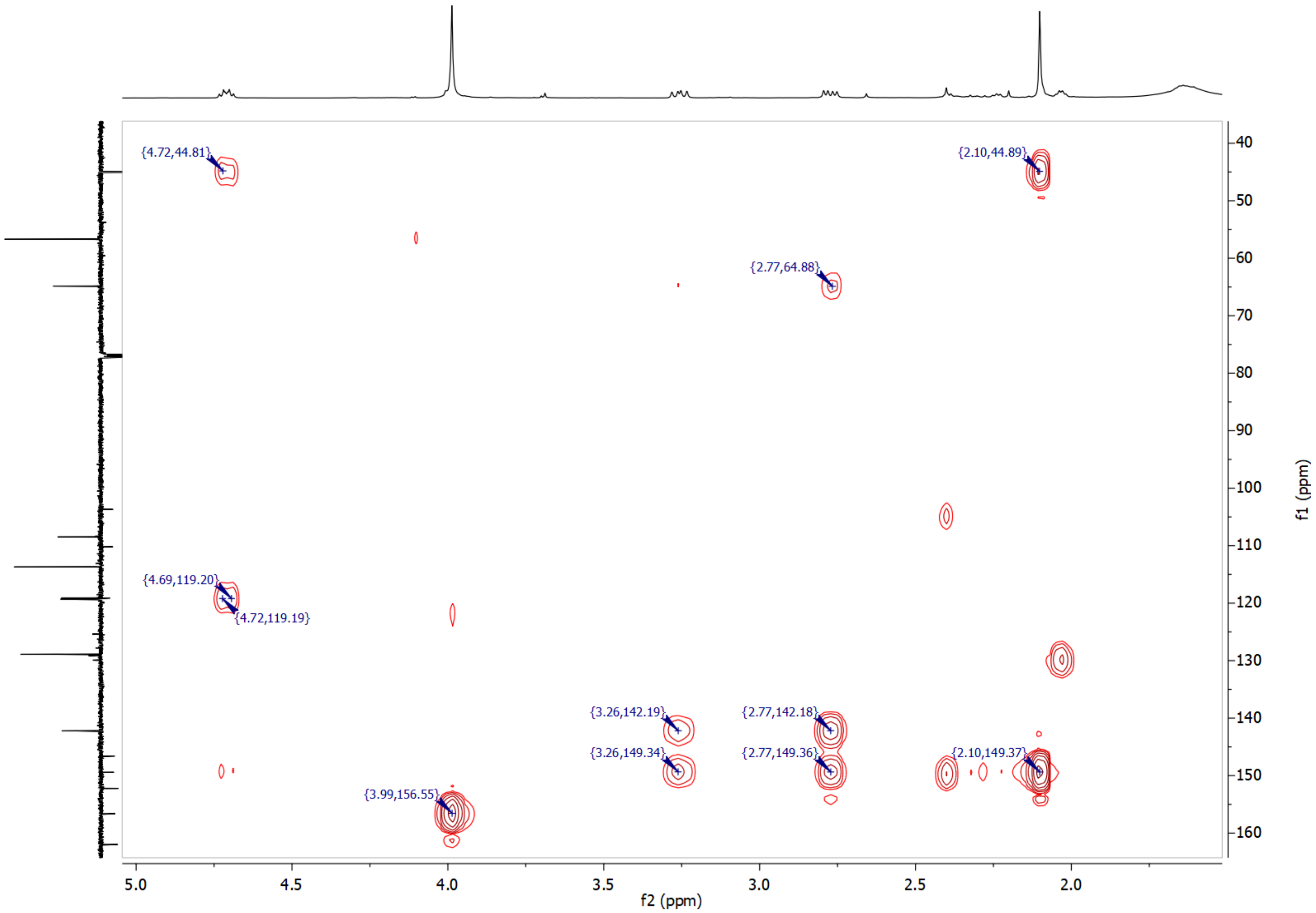

Figure S210. HMBC map for 3 from 1.0 to 5.0 ppm in $CDCl_3$.

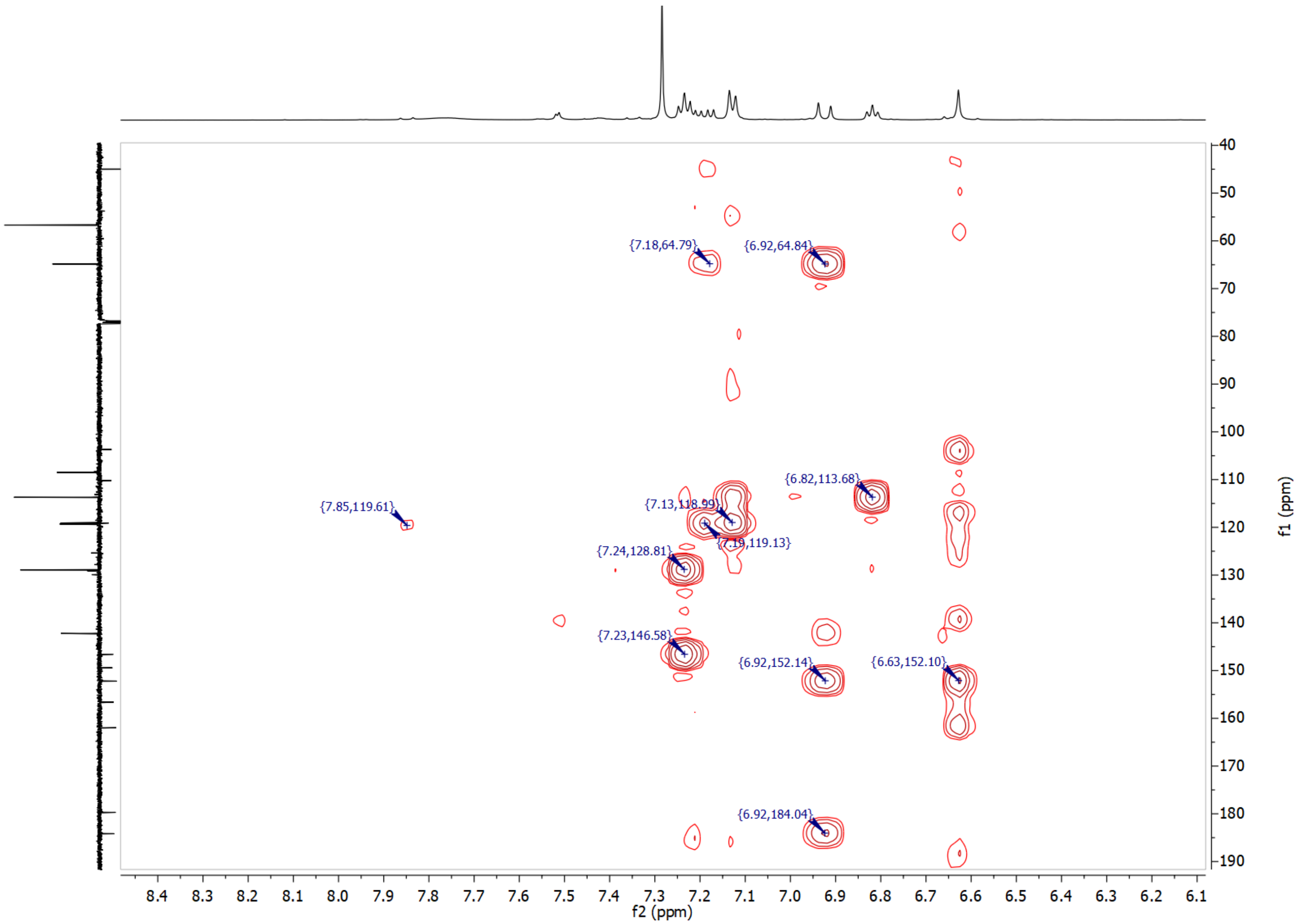

Figure S11. HMBC map for 3 from 6.0 to 8.5 ppm in $CDCl_3$.

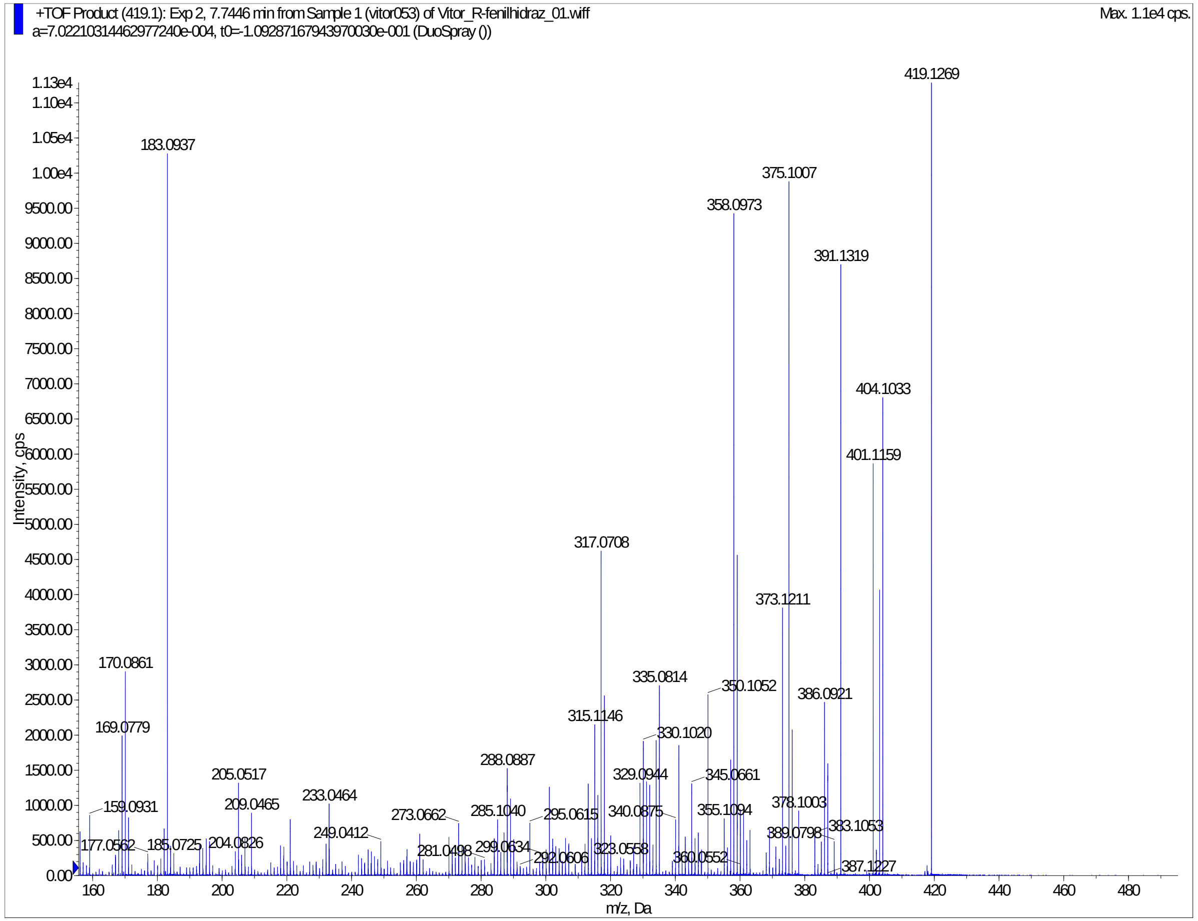

Figure S12. ESI+ CID spectra (30eV, N_2) of 4.

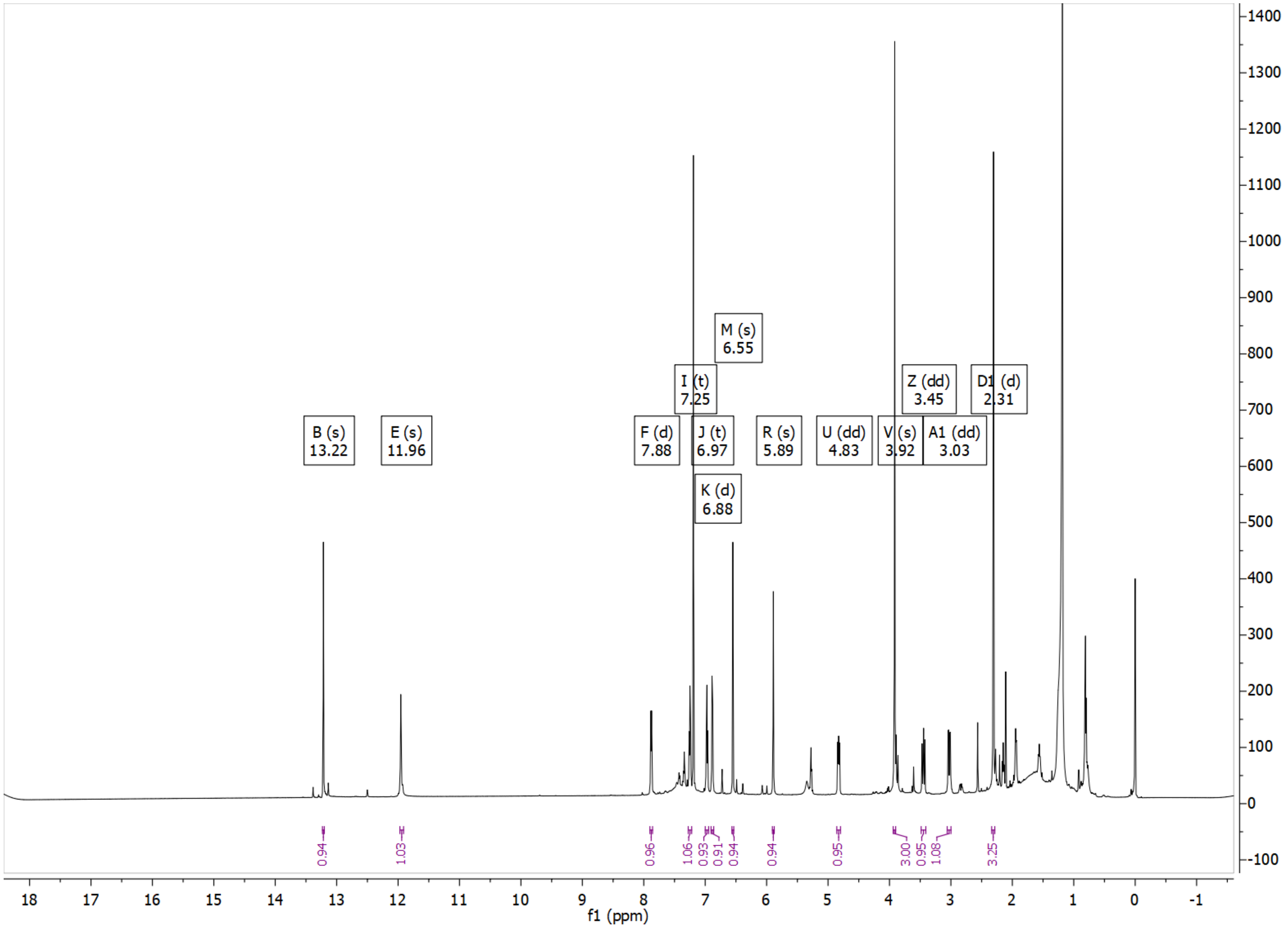

Figure S13. [1]**H NMR spectra of 4 in $CDCl_3$, 600 MHz.**

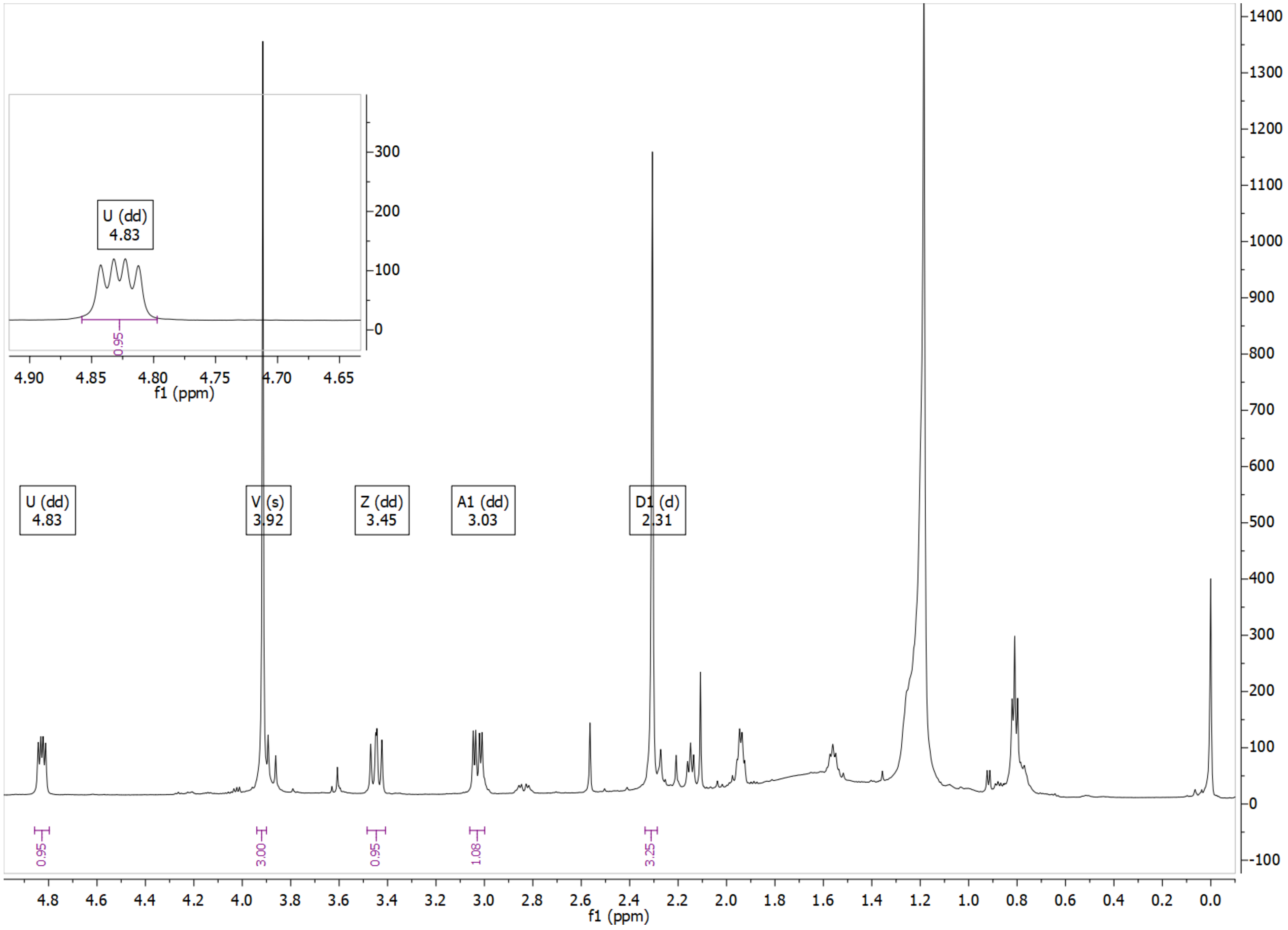

Figure S14. **^{1}H NMR spectra of 4 from 0 to 5 ppm in $CDCl_3$, 600 MHz.**

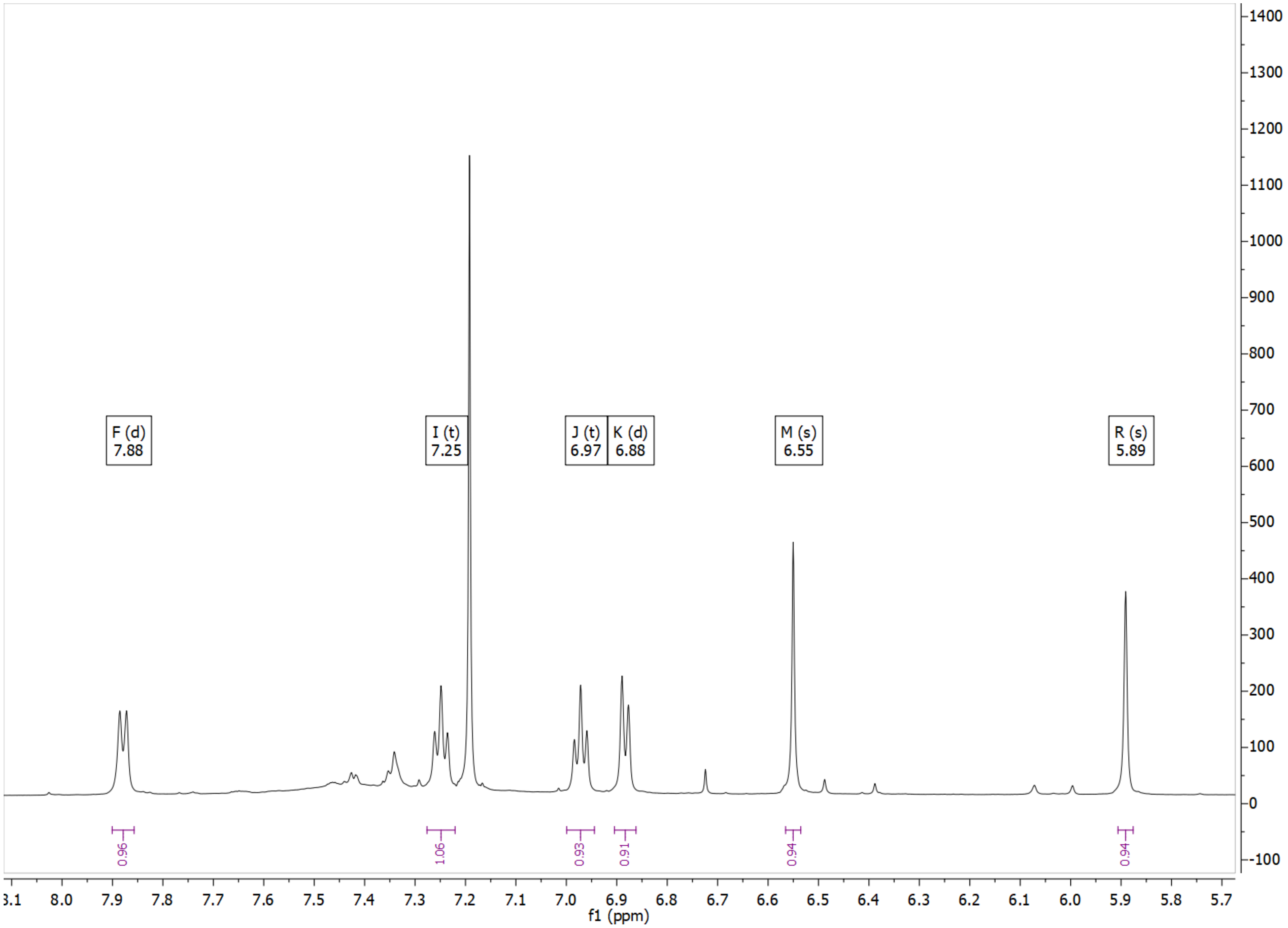

Figure S15. **^{1}H NMR spectra of 4 from 5.5 to 8.0 ppm in $CDCl_3$, 600 MHz.**

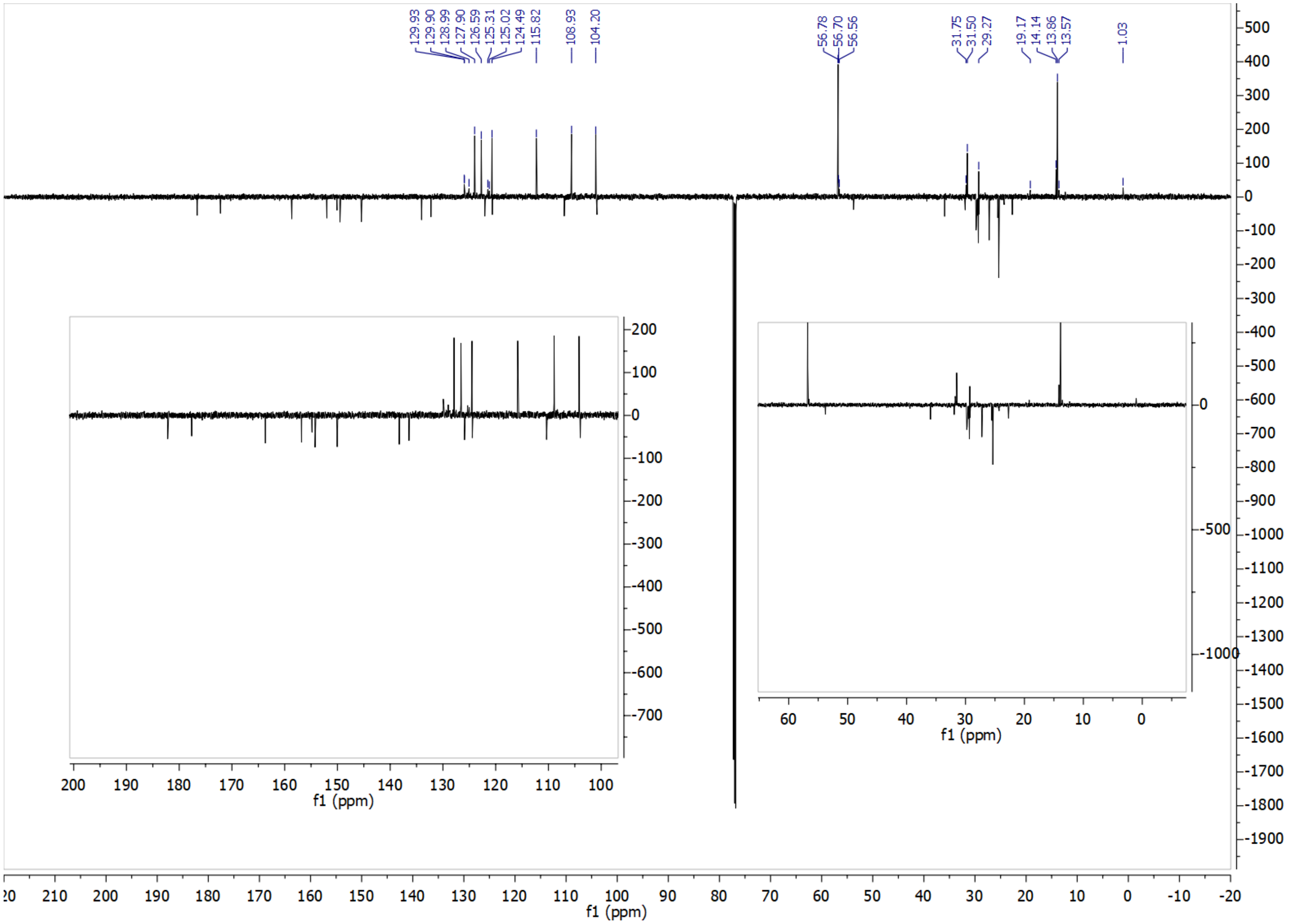

Figure S16. ^{13}C APT NMR spectra for 4 in $CDCl_3$, 150 MHz.

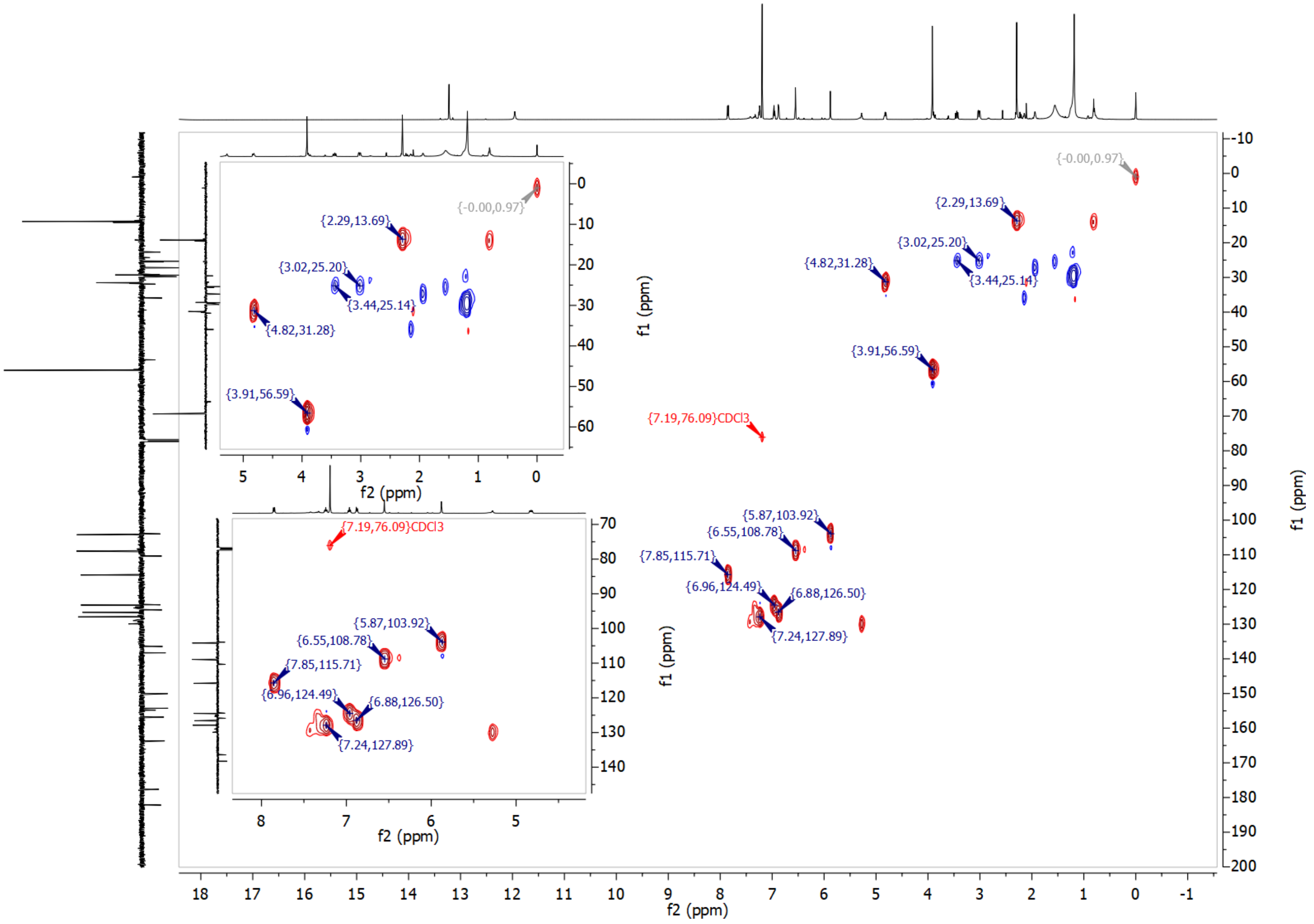

Figure S17. HMQC map for 4 in $CDCl_3$.

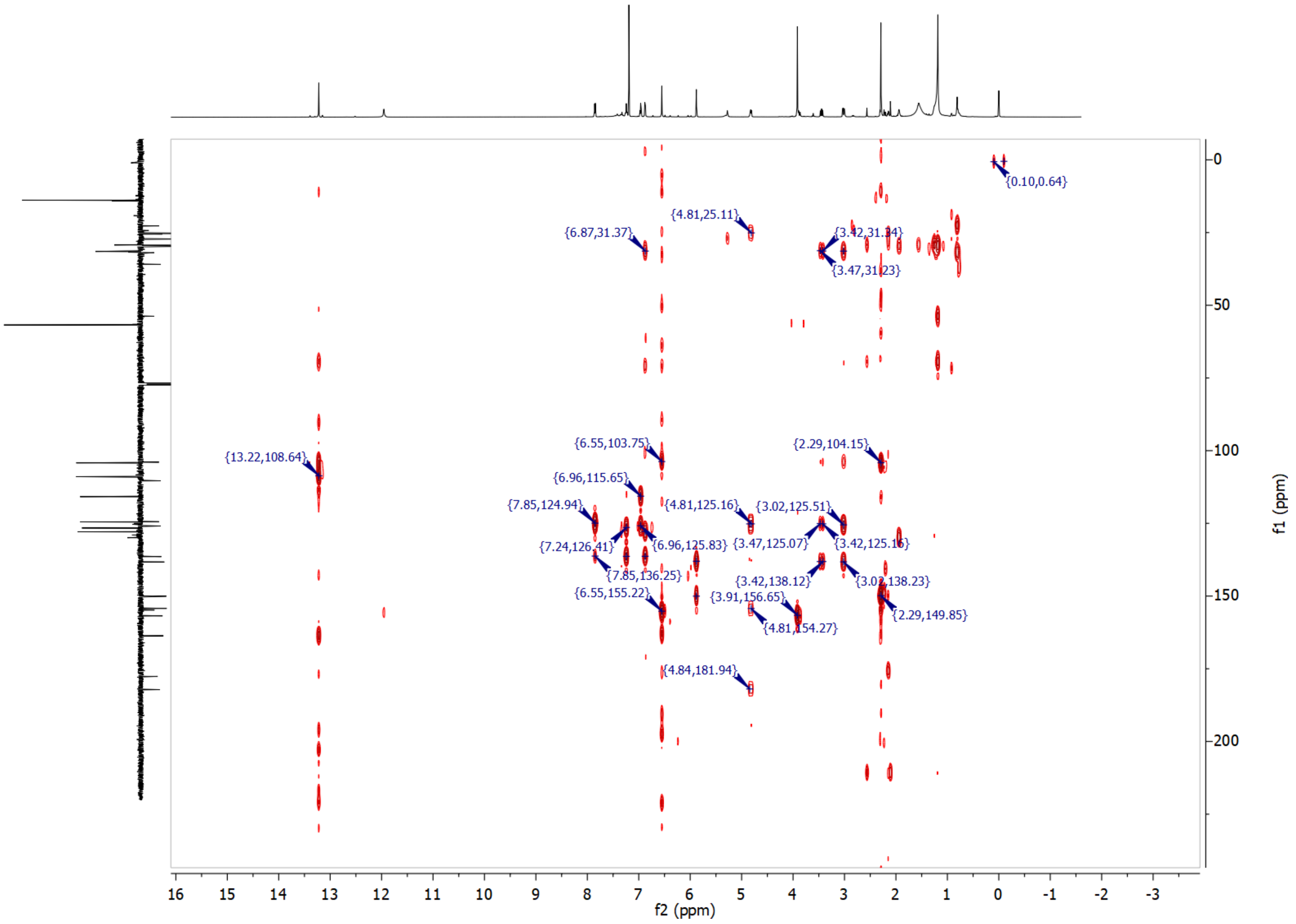

Figure S18. HMBC map for 4 in $CDCl_3$.

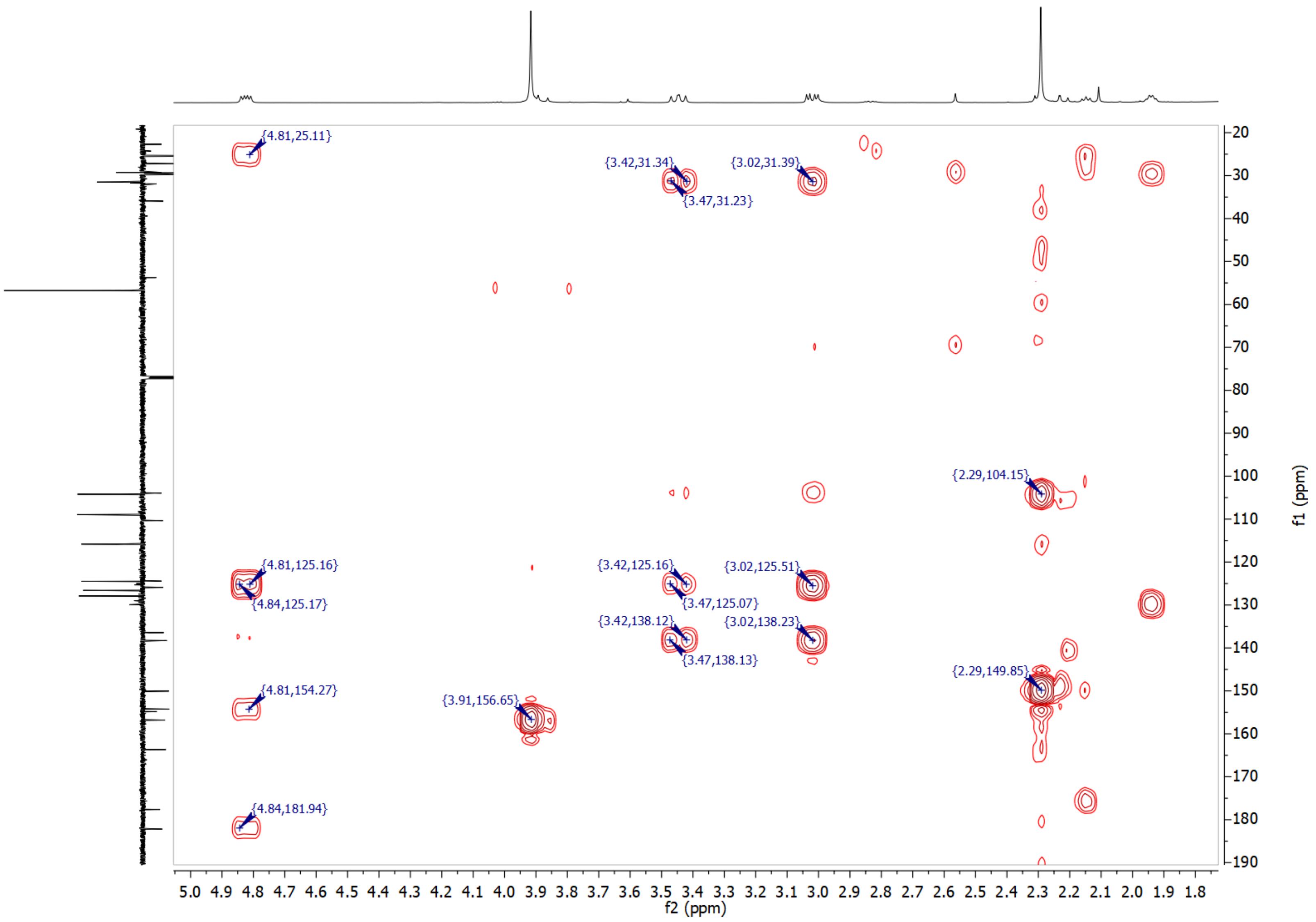

Figure S19. HMBC map from 1.5 to 5.0 ppm for 4 in $CDCl_3$.

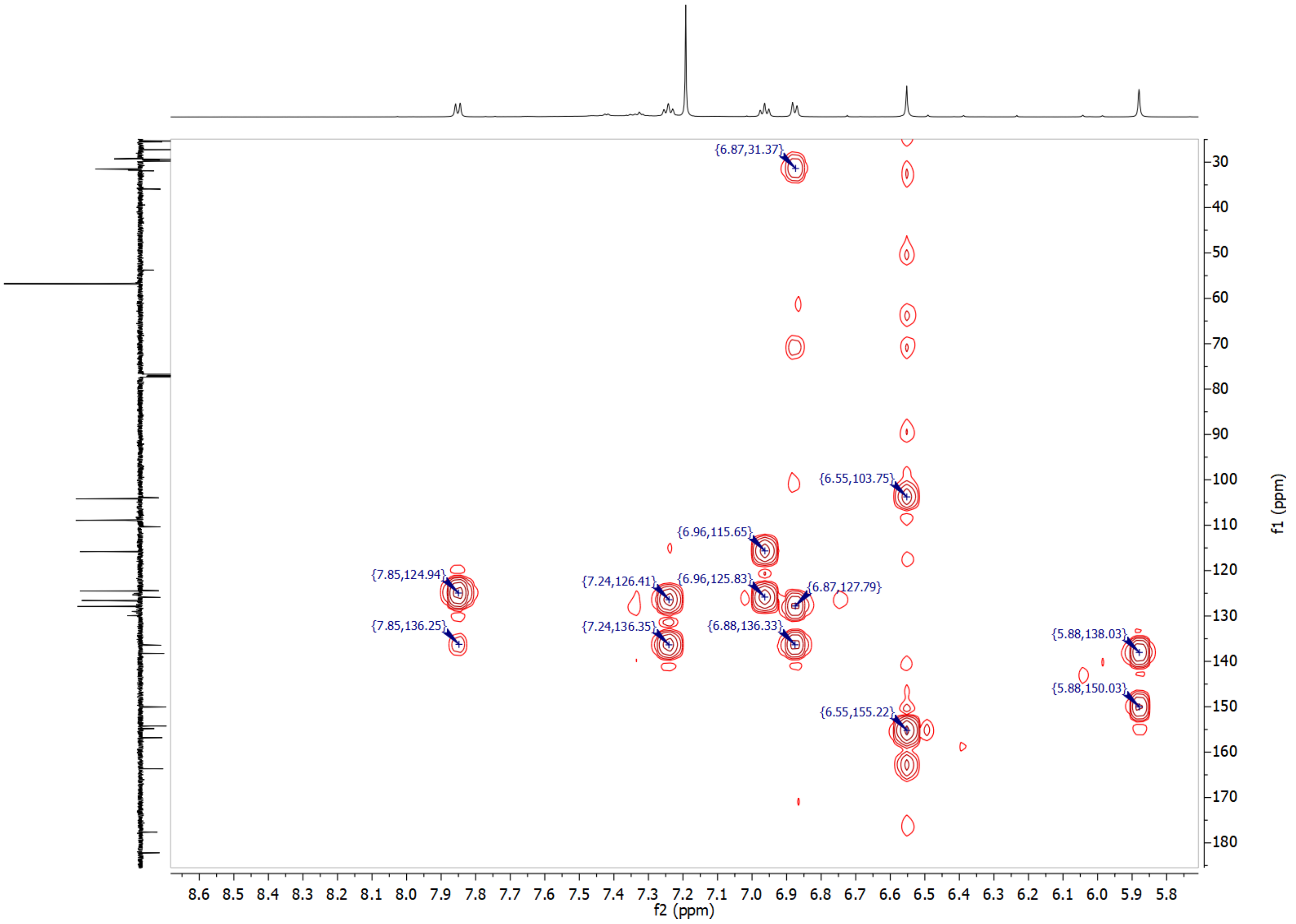

Figure S20. HMBC map from 5.5 to 8.5 ppm for 4 in $CDCl_3$.

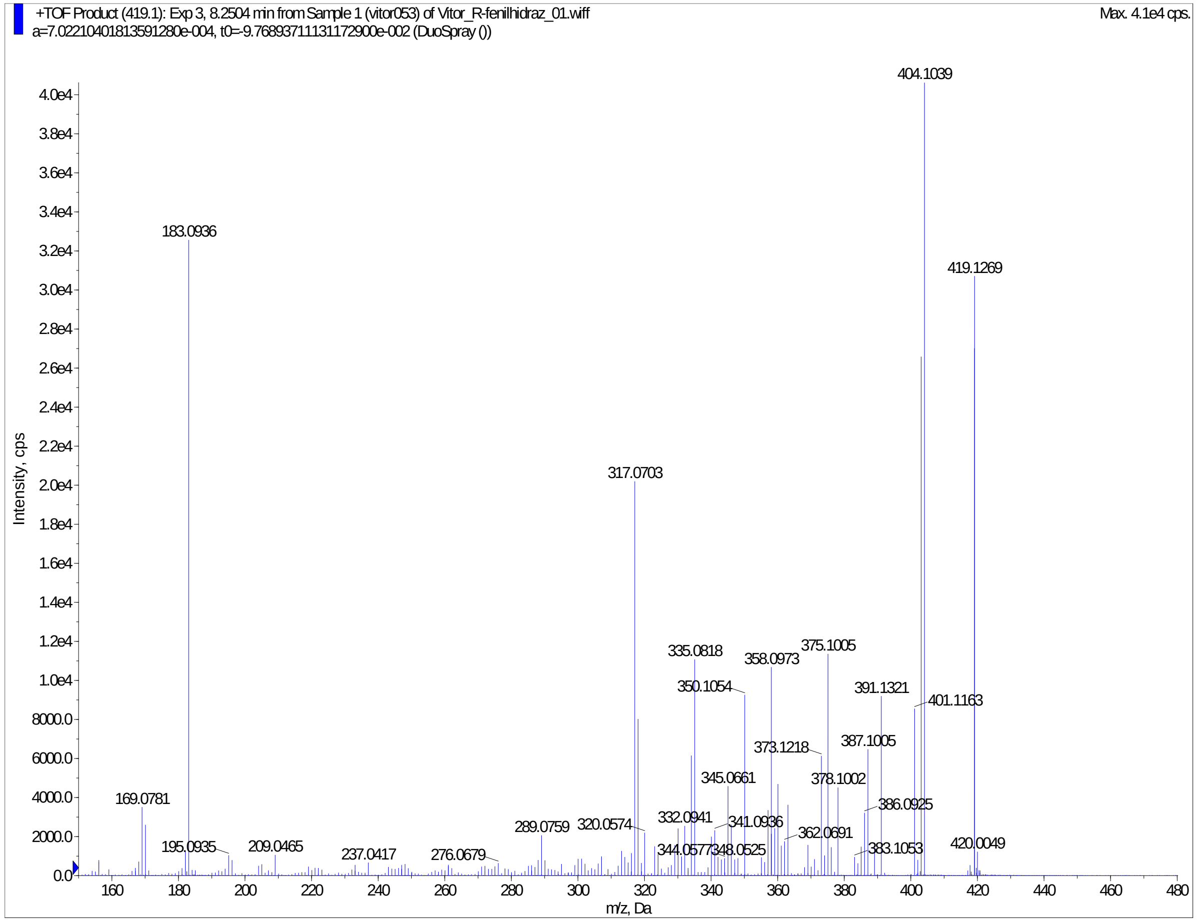

Figure S21. ESI+ CID espectra (30eV, N_2) of 5.
